# ACSL4 in Alzheimer's disease: Pathogenetic mechanisms and potential therapeutic targets

**DOI:** 10.1016/j.gendis.2025.101858

**Published:** 2025-09-18

**Authors:** Yu Guo, Qingqing Jiang, Zhongya Gu, Huan Cao, Chengchao Zuo, Yaqi Huang, Yu Song, Xiang Chen, Furong Wang

**Affiliations:** a Department of Neurology, Tongji Hospital, Tongji Medical College, Huazhong University of Science and Technology, Wuhan, Hubei 430030, China; b School of Nursing, Tongji Medical College, Huazhong University of Science and Technology, Wuhan, Hubei 430030, China; c Department of Rehabilitation, Tongji Hospital, Tongji Medical College, Huazhong University of Science and Technology, Wuhan, Hubei 430030, China; d Key Laboratory of Vascular Aging (HUST), Ministry of Education, Tongji Hospital, Tongji Medical College, Huazhong University of Science and Technology, Wuhan, Hubei 430030, China

**Keywords:** ACSL4, Alzheimer's disease, Ferroptosis, Neuroinflammation, Oxidative stress

## Abstract

Iron metabolism plays a vital role in maintaining physiological homeostasis, and its dysregulation is implicated in a range of pathological consequences and illnesses, including Alzheimer's disease (AD). Prior studies have demonstrated that Tau protein and amyloid precursor protein are involved in iron homeostasis disorder. Ferroptosis, an iron-dependent form of regulated cell death, has emerged as a key contributor to AD pathogenesis and a promising therapeutic target. Acyl-CoA synthetase long-chain family 4 (ACSL4) is a lipid metabolizing enzyme that enhances ferroptosis sensitivity by promoting the incorporation of oxidizable polyunsaturated fatty acids into membrane phospholipids. Beyond ferroptosis, ACSL4 also plays crucial roles in neuroinflammation and oxidative stress, which are implicated in AD progression. Therefore, targeting ACSL4 is fantastic and has a lot of promise for treating AD. Nevertheless, the precise mechanisms through which ACSL4 contributes to AD pathology have yet to be fully elucidated. This review reveals a potentially vital role of ACSL4 in AD, focusing on its involvement in ferroptosis, oxidative stress, and neuroinflammation. Additionally, we describe some natural and synthetic compounds targeting ACSL4 with therapeutic potential in AD. Building on the theoretical findings of earlier studies about focused interventions of the ACSL4 path, our evaluation provided a broad basis for the clinical transformation in the treatment of AD strategies.

## Introduction

Alzheimer's disease (AD) is a neurodegenerative condition characterized by progressive memory loss, aphasia, apraxia, agnosia, executive dysfunction, personality changes, and psychiatric symptoms. These manifestations greatly impede the individual's social and psychological capabilities, affecting their overall quality of life.^1^ As the most common form of dementia, AD ranks as the seventh leading cause of death globally and is among the top ten contributors to years of life lost due to disability in people aged ≥60 years.^2^ With global population aging, dementia cases are projected to rise from 78 million in 2030 to 139 million by 2050.^3^ Patients with AD frequently exhibit comorbidities, such as diabetes, hypertension, depression, stroke, and cancer, which further complicate disease management and increase healthcare burdens.

Disrupted iron homeostasis contributes to β-amyloid (Aβ) plaque and tau tangle formation in AD.^4^, ^5^, ^6^ Positron emission tomography imaging reveals elevated Aβ deposition in AD, which predicts preclinical cognitive decline.^7^ However, attempts to reduce cerebral Aβ levels through immunotherapy have not entirely halted the progression of clinical AD, implying that additional variables may exacerbate clinical deterioration.^8^^,^^9^ Current researches indicate that cortical iron buildup is a pathogenic feature of AD and elevated cerebrospinal fluid ferritin levels are correlated with poorer cognitive performance and higher AD risk.^10^^,^^11^ Ferroptosis, a regulated, iron-dependent form of cell death, has emerged as a key contributor to AD pathology. Excess iron facilitates hydroxyl radical generation through Fenton reactions, promoting lipid peroxidation, oxidative stress, and neuroinflammation.^6^ Unlike apoptosis or necroptosis, ferroptosis is driven by peroxidation of phospholipids enriched in polyunsaturated fatty acids (PUFAs).^12^ Acyl-CoA synthetase long-chain family member 4 (ACSL4) facilitates this process by converting arachidonic acid (AA) and adrenic acid (AdA) into PUFA-CoA esters, which are incorporated into phospholipids, particularly phosphatidylethanolamines (PE), rendering them susceptible to oxidation.^12^, ^13^, ^14^ In adipocyte-specific ACSL4 knockout mice, enhanced glutathione (GSH)-mediated detoxification, reduced AA incorporation into PE, and decreased 4-hydroxy-2-nonenal (4-HNE) adduct formation were observed.^13^ Although ACSL4 does not catalyze peroxidation directly, it modulates ferroptosis sensitivity by remodeling membrane lipid composition to favor oxidizable substrates.^12^, ^13^, ^14^, ^15^

As a critical modulator of lipid metabolism and ferroptosis, ACSL4 participates in multiple pathological processes, including neuroinflammation, oxidative injury, and iron dysregulation.^16^, ^17^, ^18^ Its dysfunction has been implicated in various disorders, including neurodegeneration, cardiovascular disease, cancer, and metabolic syndromes.^16^^,^^19^, ^20^, ^21^, ^22^ Elevated ACSL4 expression has been reported in aging and AD models.^23^^,^^24^ Utilizing gene expression array data from the GEO database, researchers confirmed that ACSL4 dysregulation exists in the hippocampus of AD patients.^25^ More intriguingly, SP1-ACSL4-mediated regulation of lipid peroxidation and ferroptosis was engaged in ALDH2-mediated cardio-protection in the amyloid precursor protein (APP)/presenilin-1 (PS1) mice model, and had a favorable influence on cognitive performance in AD mouse models.^26^ Suppression of ferroptosis with decreased ACSL4 alleviates memory deficits and neuronal loss in multiple AD models.^24^^,^^27^^,^^28^ Conversely, ACSL4 overexpression induces ferroptosis and promotes M2-to-M1 macrophage polarization.^29^ Upon lipopolysaccharide stimulation, ACSL4 regulates the release of pro-inflammatory factors via vestigial-like family member 4 (VGLL4) signaling.^17^ Multiple ACSL4-associated pathways, including the SystemXc–glutathione peroxidase 4 (GPX4)–ACSL4–lysophosphatidylcholine acyltransferase 3 (LPCAT3) pathway and nuclear factor erythroid 2-related factor 2 (Nrf2)/chaperone-mediated autophagy (CMA) of ACSL4 degradation, can affect neuropathological change and cognitive dysfunction in AD mice.^30^^,^^31^ Neuropsychiatric symptoms, such as anxiety and depression, are among the most difficult to treat in patients with AD. In mouse models of chronic unpredictable mild stress, fluoxetine treatment reduced ACSL4 mRNA levels and restored astrocytic and microglial function.^32^

This review summarizes previous studies on the structure, function, and regulatory mechanisms of ACSL4. It also elucidates the role of ACSL4 in AD pathogenesis, including ferroptosis, oxidative damage, and neuroinflammation, and its potential as a therapeutic target. Additionally, we discuss therapeutic strategies targeting ACSL4, including synthetic and natural compounds, providing new avenues for the development of AD therapeutic interventions.

## ACSL4 and its family members

### The structure of ACSL4

In 1997, Kang et al successfully isolated a cDNA clone that encodes a novel acyl-CoA synthetase named ACSL4.^33^ This enzyme comprises 670 amino acids and is structurally organized into five distinct regions: two luciferase-like domains (regions 1 and 2), an NH_2_ terminus, a linker region connecting the luciferase-like domains, and a COOH terminus. Among ACSL family members, the luciferase-like domain 2 and the COOH terminus show the highest sequence conservation, underscoring their critical role in catalytic activity. The lack of the corresponding 50 amino acids at the NH_2_ terminus in ACSL4 may contribute to its unique fatty acid (FA) substrate specificity.^33^

Two ACSL4 isoforms exist in mammals: a truncated variant located in the cytosol and plasma membrane, and a longer form, containing an additional N-terminal hydrophobic domain, localized in the endoplasmic reticulum and neuronal lipid droplets.^34^ The human ACSL4 gene resides on the X chromosome and is extensively expressed in the brain, ovaries, testes, and adrenal glands. ACSL4 protein is predominantly localized to the endoplasmic reticulum, mitochondria, peroxisomes, and mitochondrial-associated membranes, suggesting roles in β-oxidation and FA synthesis^35^, ^36^, ^37^ (Table 1). In addition, its association with AD is summarized in Table 2.

**Table 1:** Substrate preferences, tissue distribution, and subcellular localization of ACSL family members.

ACSL family members	Substrate preferences	Tissue distribution	Subcellular localization	References
ACSL1	PA, OA, and LA	Liver, heart, adipose tissue, and skeletal muscle	Mitochondria and endoplasmic reticulum	55, 56, 57, 58, 59, 60
ACSL3	PA, OA, AA, and EPA	Brain, testes, prostate, and skeletal muscle	Golgi, endoplasmic reticulum, mitochondria, and lipid droplets	35,36,61
ACSL4	AA and AdA	Adrenal gland, testis, ovary, and brain	Endoplasmic reticulum, mitochondria, and peroxisomes	35,66,240
ACSL5	PA, POA, OA, and LA	Intestinal mucosa, liver, lung, kidney, and adrenal gland	Mitochondria and cytoplasm	63,240
ACSL6	DHA and EPA	Brain, bone marrow, and muscle tissues	Mitochondria, cytoplasm, and membrane	66,68,241

Note: PA, palmitic acid; OA, oleic acid; LA, linoleic acid; AA, arachidonic acid; EPA, eicosapentaenoic acid; AdA, adrenic acid; POA, palmitoleic acid; DHA, docosahexaenoic acid.

**Table 2:** The regulatory mechanisms and results of ACSL family members in various Alzheimer's disease (AD) models.

ACSL family members	AD model	Regulatory mechanisms	Results	Reference
ACSL1	AD patients with the APOE4/4 subtype	ACSL1 high	Increased lipid droplet accumulation, amyloid plaque, and Tau pathology; decreased cognitive performance	172
	fAβ-challenged iMGs	ACSL1 high	Regulated lipid droplet deposition and induced pTau and apoptosis in neurons	172
	early-stage AD patients	ACSL1 high	Promoted neuronal activity, synaptogenesis, and neurogenesis while avoiding neuroinflammation and death	242
	AD patients in females	ACSL1 high	Associated with sex differences in AD susceptibility	243
	AD patients	ACSL1 high	Associated with AD risk	244
ACSL3	3xTg-AD	ACSL3 low; BDNF↓ and VEGF-C↓	AD-related anxiety and depression	245
	BPDE-induced hippocampal neurons	ACSL3 low	Induced ferroptosis	246
ACSL4	5x FAD	GPR119; NRF2	Expedited Aβ burden and cognitive deficits	23
	APP/PS1	ALDH2; SP1	Generated cognitive deficits and cardiac dysfunction	26
	SAMP8	ACSL4 high	Generated Aβ burden and cognitive deficits	24
	APP/PS1	ACSL4 high; DJ-1; Nrf2	Accelerated Aβ deposit, neuronal loss, synaptic damage, and cognitive deficits	247
	APP/PS1	AMPK/Sp1/ACSL4	Promoted cognitive deficits	248
	AD patients	hsa-mir-34a-5p and has-mir-106b-5p	High diagnostic value of the predictive model	182
	APP/PS1	ACSL4 high	Aβ accumulates in brain tissue due to lipid peroxidation	249
	Sevoflurane-induced SH-SY5Y cells death	ACSL4 high; AMPK/mTOR	Induced postoperative cognitive dysfunction	250
	Aluminum chloride-induced AD rat model	ACSL4 high; Nrf2/HO-1	Promoted Aβ and Tau accumulation; memory and learning disabilities	251
	Hippocampus in AD patients	ACSL4 low	Dysregulated ferroptosis and immune cell infiltration	25
	APP/PS1	ACSL4 high	Could be a potential biomarker of AD	235
	L-Glu-induced SH-SY5Y cells	ACSL4 high	Promoted oxidative damage and ferroptosis	252
	Aβ oligomers-induced mice	ACSL4/cPLA2	Amplified Aβ oligomer neurotoxicity and destroyed cognitive capacity	191
	APP/PS1	System Xc/GPX4/ACSL4/LPCAT3	Promoted cognitive problems	30
	Aβ_25-35_ induced-PC12 cell	ACSL4 high	Oxidative damage and ferroptosis	27
	RSL3-induced HT22 cell	ACSL4 high	Promoted ferroptosis	253
	Aβ_1–42_-induced rat synaptosomes	ACSL4 high	Generated ferroptosis and cell damage	254
	Erastin-treated SH-SY5Y cells	ACSL4 high	Induced ferroptosis and increased Aβ production	28
	BDE-47 treated mice	Nrf2-Chaperone	Brain injury and neurobehavioral disorders	31
ACSL5	–	–	–	–
ACSL6	APP/PS1	ACSL6 high	Involved in dysregulated ferroptosis	25
	AD patients	ACSL6 low; involved in a polyunsaturated fatty acid metabolism	Involved in dysregulated ferroptosis and immune cell infiltration	25

The symbol “–” indicates that no relevant studies or findings have been reported to date for this category.

### The biological functions of ACSL4

**Figure 1:**
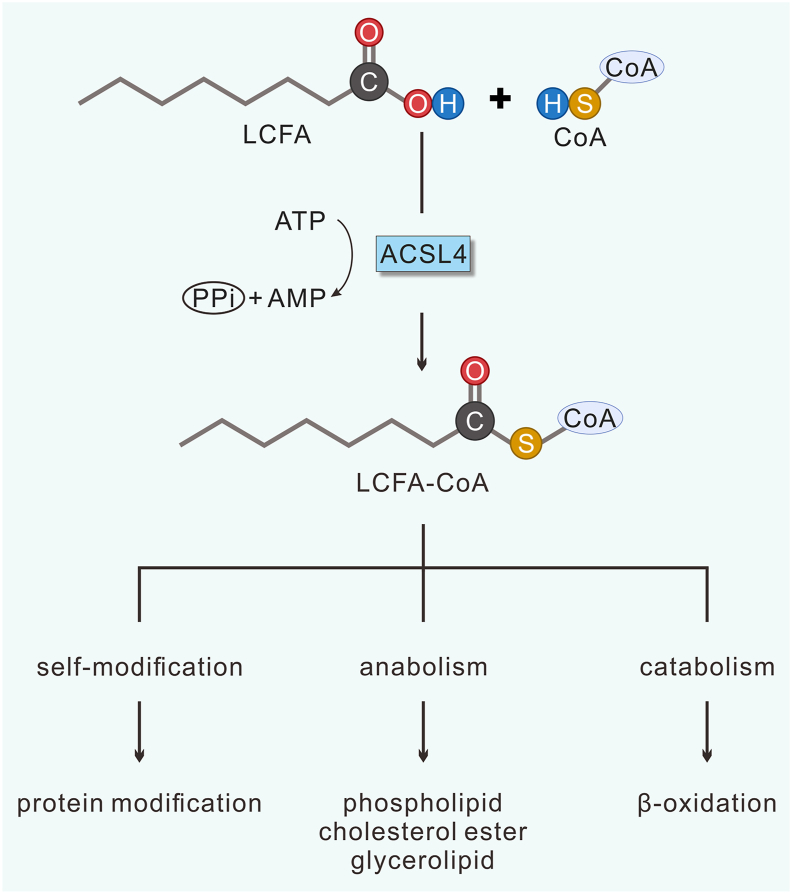
ACSL4-catalyzed reaction and the metabolic fate of products. ACSL4 catalyzes the formation of LCFA-CoA from LCFA, utilizing CoA as a cofactor and using ATP to produce AMP. Subsequently, LCFA-CoA is involved in several metabolic processes. LCFA-CoA, long-chain fatty acyl-coenzyme A; LCFA, long-chain fatty acyl; ATP, adenosine triphosphate; AMP, adenosine monophosphate; CoA, coenzyme A; PPi, pyrophosphate.

ACSL4 catalyzes the conversion of long-chain fatty acids (LCFAs) to their CoA derivatives (LCFA-CoAs). LCFA-CoA is subsequently involved in self-modification, including elongation, desaturation, and protein acylation. In addition, it is integral to both anabolic processes (biosynthesis of glycerolipid, phospholipid, and cholesterol ester) and the catabolic pathways of β-oxidation (Fig. 1).

**Figure 2:**
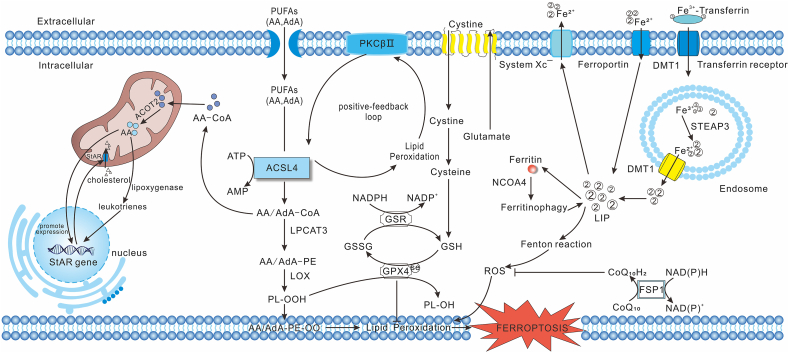
ACSL4 in ferroptosis. ACSL4 catalyzes the esterification of PUFAs, such as AA and AdA, into their CoA derivatives, which are subsequently incorporated into PEs via LPCAT3. These AA/AdA-PEs undergo oxidation by LOX to form PLOOH, a critical step driving lipid peroxidation and ferroptosis. Concurrently, iron homeostasis is regulated through transferrin-mediated uptake, ferritin storage, and NCOA4-dependent ferritinophagy, contributing to the LIP and Fenton reaction-derived ROS generation. Antioxidant systems, including GPX4–GSH and FSP1–CoQ10 pathways, act to neutralize LPOs and inhibit ferroptosis. ACSL4 also contributes to AA metabolism and leukotriene biosynthesis. Additionally, ACSL4 interacts with lipid metabolism regulators, such as PKCβII, forming positive-feedback loops to further amplify lipid peroxidation and ferroptosis signaling. PUFAs, polyunsaturated fatty acids; ACSL4, acyl-CoA synthetase long-chain family member 4; LOX, lipoxygenase; AA, arachidonic acid; AdA, adrenic acid; AA/AdA-CoA, arachidonic/adrenic acid–coenzyme A; LPCAT3, lysophosphatidylcholine acyltransferase 3; AA/AdA-PE, arachidonic/adrenic acid–phosphatidylethanolamines; PLOOH, phospholipid hydroperoxide; PL-OH, phospholipid alcohol; AA/AdA-PE-OO•, arachidonic acid/adrenic acid-phosphatidylethanolamines-peroxyl radicals; LPOs, lipid peroxides; GPX4, glutathione peroxidase 4; GSH, glutathione; GSSG, oxidized glutathione; GSR, glutathione-disulfide reductase; System Xc^−^, cystine/glutamate antiporter; ROS, reactive oxygen species; FPN, ferroportin; LIP, labile iron pool; STEAP3, six-transmembrane epithelial antigen of prostate 3; DMT1, divalent metal transporter 1; FSP1, ferroptosis suppressor protein 1; CoQ_10_, coenzyme Q10; CoQ_10_H_2_, ubiquinol; NCOA4, nuclear receptor coactivator 4; ACOT2, acyl-CoA thioesterase 2; StAR, steroidogenic acute regulatory protein; PKCβII, protein kinase C beta II.

ACSL4 plays essential roles in steroidogenesis, particularly in adrenal and Leydig cells.^38^, ^39^, ^40^ Working in concert with mitochondrial acyl-CoA thioesterase 2 (ACOT2), ACSL4 regulates mitochondrial AA release and promotes the steroidogenic acute regulatory (StAR) protein expression—a key regulator of cholesterol translocation for steroid biosynthesis.^37^^,^^41^, ^42^, ^43^ In a nutshell, intracellular free AAs are translocated into the mitochondrial compartment via the ACSL4/DBI/TSPO/ACOT2 pathway, subsequently enhancing steroidogenesis by regulating the steroidogenic acute regulatory protein (StAR)^44^ (Fig. 2). The rapid turnover of ACSL4 aligns with the necessity for precise regulation of intracellular fatty acyl-CoA ester concentrations, which are vital mediators in lipid metabolism and signaling pathways.^41^ As well, ACSL4 deficiency significantly alters membrane lipid composition, particularly reducing PE, phosphatidylcholine (PC), and phosphatidylglycerol (PG) levels.^17^ Its up-regulation enriches PUFA-containing lipids within membrane structural modifications, rendering the membrane more flexible and fluid.^45^ Beyond the above metabolic functions, ACSL4 is involved in the modulation of prostaglandin E_2_ (PGE_2_) release by human arterial smooth muscle cells,^46^ the regulation of axonal transport of synaptic vesicles and synaptic growth in drosophila melanogaster,^47^ ferroptosis-mediated 5-hydroxyeicosatetraenoic acid (5-HETE) production,^48^ and zebrafish embryogenesis.^49^

As a regulator of β-oxidation, ACSL4 facilitates LCFA mitochondrial entry via CoA conjugation and subsequent β-oxidation for energy production.^50^^,^^51^ Further studies are warranted to elucidate the interaction between ACSL4 and other β-oxidation enzymes, and to determine how ACSL4 dysfunction alters β-oxidation in diseases such as AD.

### Other members of the ACSL family

Acyl-CoA synthetases (ACSs) are divided by carbon chain length of the catalyzed FA: short-chain, medium-chain, long-chain, very long-chain, and bubblegum-type ACSs.^35^^,^^52^^,^^53^ Among the synthases, the ACSL family in mammals comprises five members, ACSL1 and ACSL3–6, which exhibit variation in tissue distributions, substrate preferences, cellular localizations, and associations with AD (Table 1, Table 2). Notably, different ACSLs have substrate overlap to a certain degree, which reflects the redundancy and compensatory role of ACSLs in LCFA metabolism.^54^

The ACSL1 gene, located on human chromosome 4q35.1, is extensively expressed in the liver, heart, adipose tissue, and skeletal muscle.^55^ ACSL1 primarily localizes to mitochondria and the endoplasmic reticulum. Normally, palmitic acid (PA), oleic acid (OA), and linoleic acid (LA) were used as substrates of ACSL1. ACSL1 functions as a crucial enzyme in mediating fatty acid oxidation (FAO), the esterification and storage of triglycerides, as well as the uptake of FAs.^56^, ^57^, ^58^, ^59^ Its localization had a dependency on TANK-binding kinase 1 (TBK1). Without TBK1, ACSL1 is shifted to the endoplasmic reticulum to promote FA re-esterification; with TBK1, it generates LCFA-CoAs for β-oxidation in mitochondria.^60^

The ACSL3 gene, located on human chromosome 2, exhibits high expression levels in the brain, testes, and skeletal muscle.^35^ Substrate preference for ACSL3 is mainly PA, OA, AA, and eicosapentaenoic acid (EPA). It predominantly localizes to the endoplasmic reticulum, mitochondria, lipid droplets, and Golgi apparatus,^35^^,^^36^^,^^61^ playing roles in regulating lipid droplet formation, autophagy, and ferroptosis.^35^^,^^36^^,^^62^

The ACSL5 gene, located on human chromosome 10, is expressed in the intestinal mucosa, liver, lung, kidney, and adrenal gland.^63^ ACSL4 prefers PA, LA, OA, and palmitoleic acid as substrates and localizes to mitochondria and cytoplasm generally. The activation of ACSL5 enhances the production of ceramide and facilitates cellular sensitivity to pro-apoptotic signals by promoting the production of the mitochondrial death protein, heat shock protein A9 (HSPA9).^63^^,^^64^ Additionally, it also regulates FA absorption and triglyceride synthesis.^65^, ^66^, ^67^

The ACSL6 gene, situated on human chromosome 5, is mainly expressed in the brain, bone marrow, and muscle tissues^68^ and is also recognized as an insulin-regulated gene.^69^ ACSL4 mediates docosahexaenoic acid enrichment in the brain 68. The neuroprotective properties of docosahexaenoic acid are ascribed to its antioxidant capacity, its enhancement of membrane fluidity, and its function as a precursor to specific pro-resolving mediators that attenuate inflammation.^70^, ^71^, ^72^, ^73^

## The regulatory mechanism of ACSL4

Emerging evidence supports that ACSL4 is precisely regulated by protein posttranslational modifications and epigenetic modifications.^74^, ^75^, ^76^, ^77^ Epigenetic regulation, such as DNA methylation, non-coding RNAs (ncRNAs), and histone modifications, modulates gene expression in a dynamic and reversible manner without altering the DNA sequence.^78^, ^79^, ^80^ Posttranslational modifications, including phosphorylation, ubiquitination, SUMOylation, and acetylation, regulate protein localization, activity, and interactions through covalent modification of specific residues.^81^^,^^82^ Although epigenetic alterations and posttranslational modifications have been linked to neurodegenerative disorders, their involvement in AD remains insufficiently characterized.^83^ Deciphering the epigenetic control of ACSL4 may yield novel biomarkers and therapeutic targets for AD prevention and intervention.

### DNA methylation

DNA methylation is the most stable epigenetic modification that regulates the transcriptional plasticity of mammalian genomes.^84^^,^^85^ This process is mediated by a class of conserved enzymes, DNA methyltransferases (DNMTs), which are responsible for adding a methyl group to position 5 of the cytosine pyrimidine ring in the CpG dinucleotide.^86^ DNMTs were initially investigated in some kinds of cancer^87^^,^^88^; however, research interests in the neuropsychiatric field have been enhanced recently.^89^, ^90^, ^91^ Studies have revealed that experience-mediated DNA methylation is required for the formation of recent memory as well as the maintenance of remote memory.^92^ Notably, overexpression of DNMT3a in the hippocampus can reverse spatial memory deficits in aged mice. However, a decline in global DNA methylation was found in the autopsied hippocampi of patients with AD.^92^ In tumor cells, the methylation pathway of ACSL4 is usually inhibited, leading to posttranslational modification changes or overexpression, which promotes tumor cell proliferation. Feng et al have reported that ACSL4 is methylated by coactivator-associated arginine methyltransferase 1 (CARM1) at R339, leading to increased ubiquitylation mediated by ring finger protein 25 (RNF25).^76^ In addition, N6-methyladenosine modification of RNA is a type of posttranscriptional methylation.^93^ METTL14 can induce ferroptosis in vascular smooth muscle cells during the thoracic aortic aneurysm by mediating the N6-methyladenosine modification of ACSL4 mRNA.^94^

### ncRNA

Post-transcriptional regulation of the ACSL4 gene is complex, mainly involving ncRNAs.^95^, ^96^, ^97^ ncRNAs, including microRNA (miRNA), long non-coding RNA (lncRNA), and circular RNA (circRNA), are a cluster of RNAs that do not encode functional proteins.^98^^,^^99^ Reports indicate that 70% of all identified miRNAs are expressed in the brain, where they contribute to neuronal development and differentiation, as well as synaptic plasticity.^100^ miR-130a-3p down-regulates ACSL4 and facilitates neuronal differentiation of neural stem cells via protein kinase B (Akt)/phosphatidylinositol 3-kinase (PI3K) signaling.^101^ It has also been established that ACSL4 is a target gene of miR-106b-5p.^96^ Another study showed that the SRY-box transcription factor 10 (SOX10) repressed the expression of ACSL4 and prevented ferroptosis in hippocampal neurons by binding to the promoter region of miR-29a-3p.^75^ In addition, depletion of circRNA Carm1 protects against acute cerebral infarction injuries by binding to microRNA-3098-3p to regulate ACSL4.^102^ Down-regulation of lncRNA plasmacytoma variant translocation 1 (PVT1) significantly promoted the progression of atherosclerosis mediated by miR-106b-5p/ACSL4.^103^

### Phosphorylation

Guang Lei proposed a model of a positive feedback loop involving lipid peroxidation and ferroptosis. This feedback loop is established when lipid peroxidation activates protein kinase CβII (PKCβII), which in turn further promotes lipid peroxidation by directly phosphorylating and activating ACSL4 at Thr328 during ferroptosis.^104^^,^^105^ Similar to PKCβII, phosphoenolpyruvate carboxykinase 2 (PCK2) phosphorylates ACSL4 at T679.^106^ ACSL4 can also be phosphorylated by cyclin-dependent kinase 1 (CDK1) at S447, affecting ubiquitination and degradation through the recruitment of ubiquitin protein ligase E3 component N-recognin 5 (UBR5).^107^ Moreover, AKT modulates ACSL4 by phosphorylating the T624 site, which activates ACSL4 to interact with S-phase kinase-associated protein 2 (SKP2), leading to its K63-linked polyubiquitination and subsequent proteasomal degradation.^108^

### Ubiquitination

Ubiquitination refers to the process by which ubiquitin molecules covalently bind to substrate proteins through a series of enzymatic reactions. The ubiquitination modification of ACSL is mainly catalyzed by a series of specific enzymes, including ubiquitin-activating enzyme E1, ubiquitin-conjugating enzyme E2, and ubiquitin ligase E3.^109^ Ubiquitination is an essential mechanism for dynamically regulating programmed cell death.^110^ Ubiquitination-dependent degradation of ACSL4 may play different functions in distinct types of cells. Prokineticin 2 (Prok2)^111^ and cytochrome P450 1B1 (CYP1B1) metabolite 20-HETE induce expression of F-box protein 10 (FBXO10), which promotes ACSL4 ubiquitination and degradation, thereby increasing neural tolerance to ferroptosis and preventing neuronal cell deaths.^112^ Two other E3 ubiquitin ligases, neural precursor cell expressed developmentally downregulated 4-like (NEDD4L)^113^ and Parkin 114, can also exert their anti-ferroptosis effects by ubiquitinating ACSL4, leading to a decrease in cardiomyocyte lipid peroxidation. Lipoprotein lipase (LPL) inhibition suppresses osteoclastogenesis by promoting ACSL4 ubiquitination through mechanisms involving ubiquitin-specific protease 14 (USP14).^115^ On the other hand, ubiquitination of ACSL4 can promote tumor progression. For instance, E3 ubiquitin ligases, including seven in absentia homolog 2 (SIAH2), FBXO10, and membrane-associated RING-CH-type finger 6 (MARCHF6), maintain ferroptosis resistance via ubiquitination of ACSL4 in tumor cells.^77^^,^^116^ Intriguingly, capsaicin directly binds to the Asp362 residue of ACSL4, which promotes synoviolin 1 (SYVN1)-mediated polyubiquitination of ACSL4 at Lys367, ultimately suppressing ferroptosis and accelerating tumor progression.^117^

### SUMOylation

SUMOylation is a process in which small ubiquitin-like modifier (SUMO) proteins are conjugated to lysine residues of their targets to regulate protein interaction and localization, transcription factor activity, genomic stability maintenance, and transcriptional regulation.^118^^,^^119^ Similar to ubiquitination, SUMOylation is catalyzed by an E1 activating enzyme, an E2 conjugating enzyme, and an E3 protein ligase.^120^^,^^121^ Previous studies reported that SUMOylation and ubiquitination can act either synergistically or antagonistically.^122^ Tripartite motif-containing 28 (TRIM28) catalyzed SUMOylation and attenuated K63-linked ubiquitination of ACSL4, thereby hindering optineurin (OPTN)-ACSL4 interaction and leading to inhibition of selective autophagic ACSL4 degradation. Furthermore, sumo-specific protease 3 (SENP3), identified as a deSUMOylating enzyme of ACSL4, was able to competitively counteract TRIM28-mediated SUMOylation.^123^ SENP1, as a widely investigated protease, decreased the stability of ACSL4 protein through deSUMOylation and indirectly inhibited its influence on phospholipid metabolic pathways downstream of ferroptosis.^124^ SUMO2 overexpression prevents the long-term potentiation impairments and cognitive decline in a mouse model of AD amyloid pathology.^125^

### Acetylation

Acetylation modification is a dynamically reversible posttranslational modification that occurs in histones or non-histone proteins, and is collectively regulated by acetyltransferases and deacetylases.^126^ ACSL4 acetylation is dual-functional. For instance, histone acetyltransferase 1 (HAT1) directly promotes the acetylation of ACSL4 at lysine 383, and deacetylase sirtuin 3 (SIRT3) mediates the deacetylation of ACSL4. Meanwhile, another deacetylase, histone deacetylase 2 (HDAC2), enhances ACSL4 acetylation by inhibiting the transcription of SIRT3. Acetylation of ACSL4 inhibits FBXO10-mediated K48-linked ubiquitination, resulting in enhanced protein stability of ACSL4. This modification contributes to the double-edged sword effect: malignant progression and enhanced tumor radiosensitivity by ferroptosis-sensitive properties.^127^

## Mechanisms by which ACSL4 contributes to AD pathology

### ACSL4-regulated lipid peroxidation susceptibility and ferroptosis

Ferroptosis is driven by the accumulation of intracellular ferrous iron (Fe^2+^) and the peroxidation of membrane phospholipids, especially those containing PUFAs. Among ACSL isoforms, ACSL4 is the most potent component for ferroptosis execution, as supported by CRISPR-based functional genomic screens.^14^ ACSL4 plays a pivotal role in lipid remodeling by catalyzing the conversion of PUFAs, such as AA and AdA, into their CoA derivatives. These PUFA-CoAs are subsequently incorporated into phospholipids via esterification by lysophosphatidylcholine acyltransferases (LPCATs).^37^^,^^128^ The resulting phospholipids containing PUFA are highly susceptible to oxidative metabolism pathway mediated by lipoxygenases (LOXs), cytochrome P450 oxidoreductases (CYPs), or phosphatidylethanolamine-binding protein 1 (PEBP1). LOXs, in particular, promote the formation of phospholipid hydroperoxides (PLOOH) and phospholipid peroxyl radicals (PLOO•), driving lipid oxidative damage.^129^^,^^130^ Accumulation of these lipid peroxides (LPOs) compromises membrane integrity, disrupts intracellular transport and signaling, and culminates in cellular dysfunction and tissue injury.^131^ As a catalyst, Fe^2+^ further participates in generating reactive oxygen species (ROS) through the Fenton reaction. Iron regulation in the brain is tightly controlled through coordinated import and export mechanisms. Iron import occurs via transferrin (TF)-bound iron and non-TF-bound iron, while export includes the transport of iron via ferroportin (FPN) to the extracellular space, its storage in ferritin, or its uptake into intracellular endocytosed vesicles before release into the cytoplasm via six-transmembrane epithelial antigen of prostate 3 (STEAP3)/divalent metal transporter 1 (DMT1). Intracellularly, excess iron is sequestered within labile iron pools (LIP)^132^, ^133^, ^134^ (Fig. 2). However, up-regulation of transferrin receptor protein 1 (TfR1) and nuclear receptor coactivator 4 (NCOA4)-mediated ferritinophagy, or FPN dysfunction, can increase LIP levels and the risk of ferroptosis.^132^, ^133^, ^134^

To counteract ferroptosis, cells rely on two main defense pathways: the GPX4–GSH and ferroptosis suppressor protein 1 (FSP1)–coenzyme Q10 (CoQ10) pathways. GPX4 is the only known enzyme that directly reduces membrane-bound PLOOH to the corresponding nontoxic phospholipid alcohol (PLOH). Typically, PLOOH can decompose into hazardous derivatives that generate covalent electrophilic compounds, which exhibit profound cytotoxic effects on biomacromolecules, such as DNA, lipids, and proteins.^135^, ^136^, ^137^ GSH is not only an important antioxidant but also an essential cofactor for the function of selenoprotein GPX4. Cystine uptake by substituting intracellular glutamate for extracellular cystine is mediated by the system Xc^−^, comprising SLC7A11 and SLC3A2 subunits, and subsequently reduced to cysteine. Glutamyl cysteine synthase utilizes cysteine, glutamate, and glycine as substrates to synthesize GSH.^138^ The FSP1–CoQ10 axis can synergize with GPX4 to mediate another protective signaling pathway.^139^ FSP1 employs NAD(P)H and CoQ10 as substrates to reduce CoQ10 to CoQ10-H2, which subsequently captures lipid radicals, particularly PLOO• and alkoxy phospholipid free radicals (PLO•), thereby reducing lipid peroxidation^140^ (Fig. 2).

Mitochondria play a complex role in ferroptosis. Mitochondrial dysfunction exacerbates ROS production and lipid peroxidation.^141^ On the other hand, β-FAO also prevents ferroptosis by degrading PUFAs, the key substrates of lipid peroxidation^142^ (Fig. 2).

### Crosstalk between ferroptosis, Aβ accumulation, and Tau phosphorylation in AD

**Figure 3:**
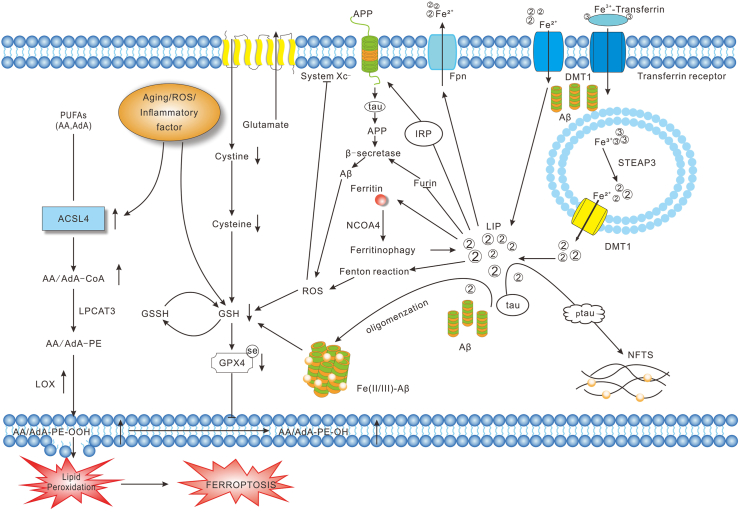
Crosstalk between ferroptosis, Aβ accumulation, and Tau phosphorylation in Alzheimer's disease. Under physiological conditions, APP exhibits ferroxidase activity, and elevated iron can be excreted with the help of APP via FPN. However, the function of APP is broken, leading to iron overload in Alzheimer's disease. Excess intracellular iron up-regulates APP through increased IRP activity, while inhibiting the normal function of furin, resulting in the up-regulation of β-secretase, thus accelerating the expression and deposition of Aβ. Oxidative stress, aging, and inflammation elevate ACSL4 and LOX expression while depleting GSH, promoting PUFA peroxidation and ferroptosis. Concurrently, increased Aβ deposition and Tau phosphorylation disrupt GSH biosynthesis and reduce the level and detoxification capacity of GPX4, ultimately amplifying lipid peroxidation. Furthermore, redox-active Fe^2+^ accelerates Aβ oligomerization and tau phosphorylation, contributing to amyloid plaque and neurofibrillary tangle (NFT) formation, hallmarks of Alzheimer's disease pathology. APP, amyloid precursor protein; Aβ, β-amyloid; IRP, iron-regulatory protein; FPN, ferroportin; PUFAs, polyunsaturated fatty acids; ACSL4, Acyl-CoA synthetase long-chain family 4; LOX, lipoxygenase; GSH, glutathione; GPX4, glutathione peroxidase 4; LPOs, lipid peroxides; NET, neurofibrillary tangles; AA, arachidonic acid; AdA, adrenic acid; AA/AdA-CoA, arachidonic acid/adrenic acid-coenzyme A; LPCAT3, lysophosphatidylcholine acyltransferase 3; AA/AdA-PE, arachidonic acid/adrenic acid-phosphatidylethanolamines; AA/AdA-PE-OOH, arachidonic acid/adrenic acid-phosphatidylethanolamines-hydroperoxides; GSH, glutathione; GSSG, oxidized glutathione; GPx4, glutathione peroxidase 4; System Xc^−^, cystine/glutamate antiporter; ROS, reactive oxygen species; LIP, labile iron pool; STEAP3, six-transmembrane epithelial antigen of prostate 3; DMT1, divalent metal transporter 1; pTau, phosphorylated Tau.

A number of studies have confirmed that some ferroptosis inhibitors and molecular inhibitors targeting ACSL4 can alleviate pathological changes and cognitive impairment in AD mouse models by inhibiting ACSL4 activity.^30^^,^^31^^,^^137^^,^^143^^,^^144^ Butterfield and Boyd-Kimball discovered that LPOs and Aβ plaques were found to be co-localized in AD patient brains.^145^ Additionally, iron overload accelerates Aβ oligomerization and tau phosphorylation, contributing to amyloid plaque and neurofibrillary tangle formation.^146^^,^^147^ Autopsies and magnetic resonance imaging have revealed iron deposits in cortical tau accumulations and around Aβ plaques, suggesting the interaction of iron with these AD pathological features.^148^^,^^149^ Under physiological conditions, APP has ferroxidase activity and aids in iron excretion through FPN. In AD, however, the function of FPN is impaired, leading to iron retention. Furin regulates the balance of α-secretase and β-secretase activities during APP cleavage.^150^ Iron overload suppresses furin, shifting APP cleavage toward β-secretase, while also enhancing iron-regulatory protein (IRP)/iron-responsive element (IRE) interaction and increasing APP expression.^150^^,^^151^ Elevated APP is cleaved to Aβ40/42 by additional β-secreting enzymes, accelerating the deposition of Aβ and promoting iron accumulation, which forms a self-reinforcing cycle^133^^,^^152^ (Fig. 3). Additionally, the targeted knockdown of GPX4 in neurons located in the cortex and hippocampus has led to neuron degeneration and cognitive deficits in mice.^153^^,^^154^ Mutations in presenilin 1 and 2 are implicated in autosomal dominant familial AD. Presenilin mutations in AD may disrupt selenium uptake, reduce GPX4 expression, and increase cellular vulnerability to ferroptosis.^155^

### ACSL4 and oxidative stress in AD

**Figure 4:**
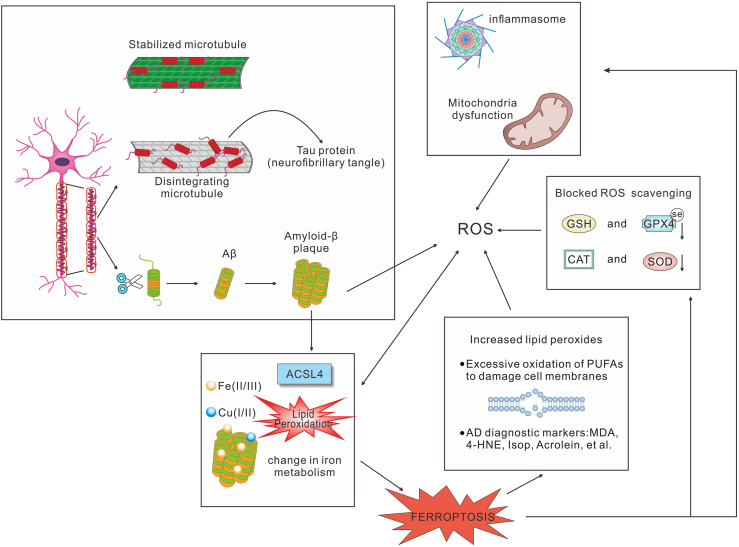
Ferroptosis exacerbates oxidative stress and neurodegeneration in Alzheimer's disease. Poor prognosis of Alzheimer's disease is associated with dysregulation of ion metabolism, particularly iron and copper, in the central nervous system. Imbalance of intracellular iron metabolism and ACSL4-mediated regulation of lipid peroxidation and ferroptosis lead to elevated lipid peroxidation products, such as 4-HNE and MDA, which will further lead to DNA, cell membrane, and mitochondrial damage. Concurrently, Aβ aggregation and tau hyperphosphorylation disrupt cytoskeletal stability and mitochondrial function, contributing to ROS accumulation. Depletion of antioxidant systems, including GPX4, GSH, CAT, and SOD, impairs ROS scavenging, further amplifying oxidative stress. Additionally, mitochondrial dysfunction and inflammasome activation act as both consequence and amplifier of ferroptosis, creating a vicious cycle of neuroinflammation and oxidative injury in Alzheimer's disease pathology. Aβ, β-amyloid; ROS, reactive oxygen species; GSH, glutathione; GPX4, glutathione peroxidase 4; CAT, catalase; SOD, superoxide dismutase; ACSL4, acyl-CoA synthetase long-chain family member 4; PUFAs, polyunsaturated fatty acids; 4-HNE, 4-hydroxy-2-nonenal; MDA, malondialdehyde; IsoPs, isoprostanes.

Dysregulated PUFA metabolism mediated by ACSL4 contributes to neurotoxicity in AD. The brain, second only to adipose tissue in lipid content, is particularly rich in PUFAs within neuronal and organellar membranes. Owing to its high oxygen consumption and PUFA abundance, the brain is more susceptible to lipid peroxidation than other organs.^141^^,^^156^^,^^157^ Mitochondrial dysfunction is a major source of ROS in the brain. Disrupted iron metabolism, impaired tricarboxylic acid cycle, and electron transport chain abnormalities lead to electron leakage, generating free radicals that attack nearby molecules.^158^ Lipid peroxidation involves oxidative attacks—both free radicals and non-free radicals—on carbon–carbon double bonds, particularly in PUFA-containing phospholipids.^159^ Neuronal membranes, rich in AA and AdA, are especially susceptible to peroxidation-induced cytotoxicity^131^ (Fig. 4).

Ferroptosis not only leads to cell death but also is an amplifier of the oxidative chain. AA and AdA are catalyzed and oxidized stepwise by ACSL4 and other enzymes, with overloaded iron reacting via the Fenton reaction, to produce reactive aldehyde and LPOs, which eventually amplify the oxidative chain reaction and accelerate the progression of AD.^30^ The abundant diffusible aldehyde and LPOs, such as malondialdehyde (MDA), acrolein, 4-hydroxy-2-hexenal (4-HHE), and 4-HNE,^129^^,^^130^^,^^160^^,^^161^ induce oxidative damage mainly by interacting with DNA and the residues of cysteine, lysine, and histidine on proteins.^160^^,^^162^ Increased levels of 4-HNE and acrolein are negatively correlated with cognitive function in the brains of those with mild cognitive impairment and early AD.^163^, ^164^, ^165^ Chen et al developed a new sporadic AD animal model by injecting acrolein, an endogenous aldehyde, into C57BL/6 mice and found that this model caused cognitive deficits and AD-like pathological changes, such as the accumulation of Aβ1-42 and p-Tau, glial cell proliferation, and synaptic dysfunction in the cerebral cortex and hippocampus.^166^ Isoprostanes (IsoPs) and isofurans (IsoFs), stable oxidative metabolites of AA, are elevated in brain lesions of late-stage AD patients, while early-stage patients show increased IsoPs in cerebrospinal fluid^167^ (Fig. 4).

In addition, decreased ferroptosis regulators, such as GSH and GPX4, further impair redox balance and block ROS clearance in AD^168^^,^^169^ (Fig. 4). Notably, ACSL4-deficient cells show greater resistance to ferroptosis induced by GPX4 inhibitors compared with SLC7A11 inhibitors,^170^ suggesting that ACSL4 in ferroptosis is closely related to GPX4-GSH depletion.

### ACSL4 and neuroinflammation in AD

**Figure 5:**
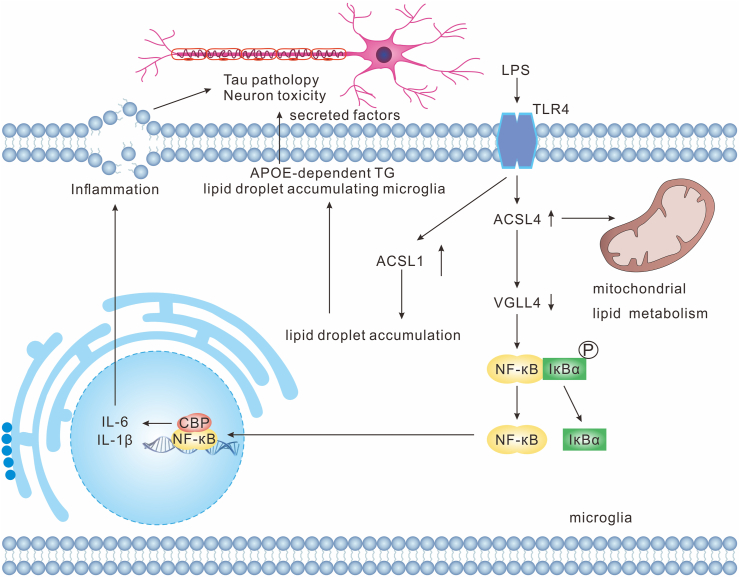
LPS-induced ACSL4 activation contributes to neuroinflammation and lipid dysregulation in microglia associated with Alzheimer's disease. Upon LPS stimulation, microglial ACSL4 expression is up-regulated, which suppresses the production of VGLL4 and enhances NF-κB signal, resulting in the production of pro-inflammatory cytokines, such as IL-1β and IL-6. The secreted inflammatory mediators induce tau pathology and neuronal dysfunction, exacerbating neurodegeneration. In parallel, ACSL4 contributes to mitochondrial lipid metabolic remodeling. Additionally, ACSL1 regulates lipid droplet (LD) accumulation, further leading to the production of tau protein in neurons, which causes lesions in the Alzheimer's brain. VGLL4, vestigial-like family member 4; ACSL4, Acyl-CoA synthetase long-chain family 4; ACSL1, Acyl-CoA synthetase long-chain family 1; NF-κB, nuclear factor kappa-B; IκBα, inhibitor of NF-κB alpha; LPS, lipopolysaccharide; IL-6, interleukin-6; IL-1β, interleukin-1 beta; TG, triglyceride; APOE, apolipoprotein E.

ACSL4 contributes to neuroinflammation by regulating lipid metabolism and nuclear factor kappa-B (NF-κB) activation. Knockdown of ACSL4 altered the levels of certain phospholipids, such as PE, PC, and PG.^17^ NF-κB is an important transcription factor that mediates inflammation and is closely linked to microglial activation.^171^ ACSL4 activates NF-κB indirectly to promote microglia-mediated neuroinflammation by regulating lipid metabolism and VGLL4 expression. In addition, regulation of microglial inflammation by ACSL4 is independent of ferroptosis in the MPTP model^17^ (Fig. 5). Another recent study also confirmed that ACSL4 silencing in microglia reduced the release of inflammatory cytokines after oxygen and glucose deprivation, however, knockdown of ACSL4 in microglia failed to reduce lipid peroxidation, a marker of ferroptosis, after oxygen and glucose deprivation.^16^ Furthermore, microglia in AD mice exhibited a cellular state of lipid droplet accumulation and dysfunction called lipid droplet-accumulating microglia.^172^ ACSL1 is a key enzyme closely related to lipid droplet-accumulating microglia biogenesis. ACSL1-positive microglia were enriched near Aβ plaques in AD patients with APOE4/4 genotypes (inheriting two copies of apolipoprotein E4 (APOE4)), associated with cognitive decline and enhanced tau pathology.^172^ Additionally, a previous investigation in bone marrow-derived macrophages has confirmed that ACSL4 promotes the production of inflammatory cytokines by increasing the insertion of HUFAs into phosphatidylcholines.^173^ In another, myeloid cell-specific deficiency of ACSL4 decreased inflammation by remodeling phospholipids and reducing the generation of proinflammatory lipid mediators.^174^

**Figure 6:**
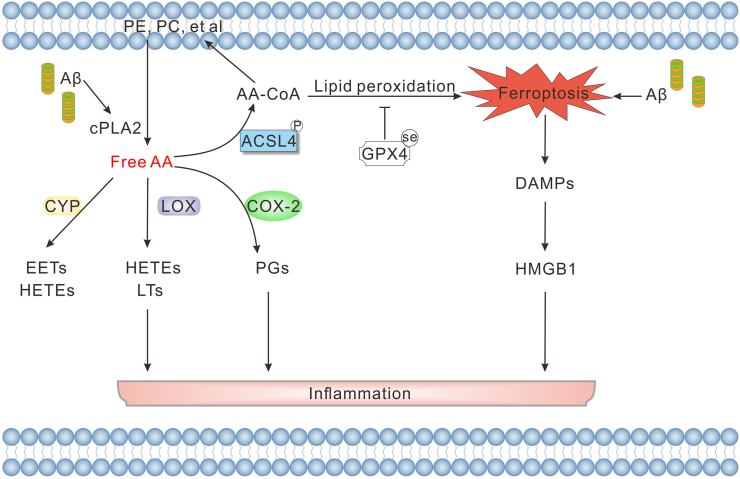
ACSL4-regulated ferroptosis contributes to neuroinflammation in Alzheimer's disease. Aβ activates cPLA2, catalyzing the release of free AA from membrane phospholipids (*e.g.*, PE and PC). Free AA is converted by ACSL4 to AA-CoA, entering lipid peroxidation pathways that promote ferroptosis. Simultaneously, free AA is metabolized by COX-2, LOX, and CYP to generate various inflammatory lipid mediators, including PGs, HETEs, and LTs, all contributing to inflammation. Ferroptosis leads to the release of DAMPs, such as HMGB1, which further perpetuate the neuroinflammatory response. ACSL4, acyl-CoA synthetase long-chain family 4; Aβ, β-amyloid; cPLA2, cytosolic phospholipase A2; AA, arachidonic acid; AA-CoA, arachidonic acid–coenzyme A; PE, phosphatidylethanolamine; PC, phosphatidylcholine; GPX4, glutathione peroxidase 4; COX-2, cyclooxygenase-2; LOX, lipoxygenase; CYP, cytochrome P450 monooxygenase; PGs, prostaglandins; HETEs, hydroxyeicosatetraenoic acids; LTs, leukotrienes; EETs, epoxyeicosatrienoic acids; DAMPs, damage-associated molecular patterns; HMGB1, high-mobility group box 1.

Ferroptosis triggered by ACSL4-driven lipid remodeling also promotes neuroinflammation. Unlike apoptosis, ferroptosis is immunogenic, and ferroptosis itself can amplify inflammation by releasing damage-associated molecular patterns (DAMPs), which further activate immune cells and perpetuate the inflammatory response^175^, ^176^, ^177^, ^178^ (Fig. 6). In addition, iron accumulation generates ROS to trigger microglial activation and increase the secretion of proinflammatory factors.^179^^,^^180^ GPX4, which reduces complex phospholipid peroxides and inhibits the activation of enzymes related to AA metabolism, plays a crucial role in limiting lipid peroxidation.^181^ Saikosaponin B2 exhibited anti-ferroptosis and anti-neuroinflammation effects through the Toll-like receptor 4 (TLR4)/NF-κB pathway in a GPX4-dependent manner in the chronic unpredictable mild stress model.^144^ A bioinformatics perspective analyzes that ACSL4 is dysregulated in the hippocampus of AD patients, which is significantly related to the functional metabolism and immune microenvironment of the patients.^182^ In another analysis, ferroptosis-related differentially expressed genes regulated the immune cell infiltration pattern in the AD hippocampus, characterized by decreased memory B cells and increased memory resting CD4^+^ T cells, memory activated CD4^+^ T cells, and resting natural killer cells.^25^ In addition, ferroptosis can also strongly induce inflammation through the release of interleukin (IL)-33 or other unidentified pathways.^176^^,^^183^^,^^184^ ACSL4-specific and ferroptosis inhibitors, such as rosiglitazone, ferrostatin-1 (Fer-1), liproxstatin-1 (Lip-1), and melatonin, are involved in inhibiting ACSL4 expression, lipid peroxidation, and ferroptosis to suppress the subsequent immune cell infiltration and inflammatory response.^29^^,^^31^^,^^144^^,^^185^^,^^186^

ACSL4 promotes inflammatory responses that may be related to the AA metabolic network. One study indicates that Lip-1, a ferroptosis inhibitor, can down-regulate the expression of ACSL4 and cyclooxygenase-2 (COX-2) simultaneously.^186^ Moreover, multiple studies have confirmed that the activation of COX also links ferroptosis and chronic inflammation.^187^ Importantly, the expression of COX2 is tightly regulated by ACSL4.^48^^,^^188^ COX-2, encoded by the prostaglandin-endoperoxide synthase 2 (PTGS2) gene, accelerates AA metabolism and amplifies inflammation by secreting inflammatory signaling molecules.^176^^,^^187^ Specifically, AA is a kind of ω-6 PUFA, and COX catalyzes free AA to form an unstable hydrogen peroxide intermediate called prostaglandin G2, which is converted to different species, including PGE2 and prostaglandin F2α (PGF2α), actively participating in the inflammatory response through the action of prostaglandin H synthase 2.^48^^,^^189^^,^^190^ In the AA-fed AD model, Aβ induced cytosolic phospholipase A2 (cPLA2) activation, and the AA released from cPLA2 can be re-embedded into the membrane by lysophosphatidyltransferase after catalytic activation of AA into AA-CoA by ACSL4, eventually leading to the neurotoxicity of expanded Aβ oligomers and cognitive impairment^191^ (Fig. 6).

## Targeting ACSL4 to treat AD

We provide an overview of natural and synthetic compounds targeting ACSL4, which has shown promising and excellent therapeutic prospects for the treatment of AD.

### Natural ACSL4 inhibitors

Melatonin, a neurohormone secreted by the pineal gland, readily penetrates the blood-brain barrier and exerts a powerful neuroprotective effect in various central nervous system disorders, including cognitive dysfunction,^192^ neurodegeneration,^193^ sleep deprivation,^194^ and depression.^195^ It can prevent long-chain PUFAs, especially reducing the loss of docosahexaenoic acid and AA, from nonenzymatic lipid peroxidation in rat brain microsomes.^196^ Additionally, it promotes the ACSL4 ubiquitination and degradation and confers neuroprotection via enhancing FBXO10^112^ or murine double minute 2 (MDM2).^197^ It also activates Nrf2 to promote CMA degradation of ACSL4, thereby ameliorating BDE-47-induced neuronal ferroptosis and cognitive deficits.^31^

Triacsin C (TC) directly inhibits ACSL4 in a dose-dependent manner by competing with FAs to bind to their catalytic domain.^26^^,^^35^^,^^198^, ^199^, ^200^ Using TC as an ACSL4 inhibitor, Zhu et al provide the first evidence that the mitochondrial ALDH2 transgene effectively protects against APP/PS1 mutation-induced cardiac atrophy, contractile dysfunction, and mitochondrial injury via SP1/ACSL4-mediated regulation of lipid peroxidation and ferroptosis.^26^ Moreover, lipid droplet-accumulating microglia represent a dysfunctional and proinflammatory state in the aging brain. TC hinders the production of lipid droplets in oxygen and glucose deprivation-induced microglia by inhibiting the *de novo* synthesis of glycerolipids and polarizes microglia towards an anti-inflammatory phenotype.^201^

Beta-hydroxybutyrate (BHB), a major constituent of ketone bodies, confers inherent neuroprotection for the immature brain.^202^ By controlling zinc finger protein 36 (ZFP36) and directly stabilizing ACSL4 mRNA, BHB reduces oxidative stress and ferroptosis of dopaminergic neurons in Parkinson's disease.^203^ Furthermore, injecting exogenous BHB can ameliorate neuronal death and lower pathology scores in hypoxic-ischemic encephalopathy.^204^

Da Chuanxiong (DCX), a traditional herbal blend of *Ligusticum chuanxiong Hort.* and *Gastrodia elata Bl.*, offers neuroprotection and enhances cerebral circulation. In AD mouse models, it efficiently regulates p-Tau protein levels to reduce cognitive decline and promote neurogenesis.^205^, ^206^, ^207^ It also mitigates lipid peroxidation and shields neurons from harm by controlling the ASCL4/GPX4-mediated ferroptosis, improving cognitive impairment related to vascular issues.^208^

Baicalin, a lipophilic flavonoid glycoside from *Scutellaria baicalensis*, exhibits anti-apoptotic and antioxidant effects.^209^, ^210^, ^211^, ^212^ In an intracerebral hemorrhage model, it alleviates motor impairments and brain damage by suppressing ferroptosis without harming the liver or kidneys.^213^ It also exerts anti-fibrotic effects by lowering ACSL4 expression through miR-3595.^214^ Notably, baicalin effectively penetrates the blood-brain barrier and improves subarachnoid hemorrhage-induced brain injury.^211^

Paeonol (PAN), derived from the *Paeonia genus*, has been shown to slow the progression of hypertension, epilepsy, and arthritis.^215^, ^216^, ^217^ Mechanistically, upstream frameshift 1 (UPF1) and ACSL4 are downstream targets of HOX transcript antisense RNA (HOTAIR). HOTAIR competes with UPF1, a factor that promotes ACSL4 degradation, thus modulating the HOTAIR/UPF1/ACSL4 axis. PAN attenuates intracerebral hemorrhage progression by regulating this axis. In hemin-treated neurons, PAN suppresses ferroptosis by targeting ACSL4, whereas HOTAIR overexpression reverses this effect.^218^

### Synthetic ACSL4 inhibitors

Thiazolidinediones, including troglitazone, rosiglitazone, and pioglitazone, have been identified as specific ACSL4 inhibitors. Thiazolidinediones effectively block ACSL4-mediated activation of PUFAs and subsequent lipid peroxidation in a manner independent of peroxisome proliferator-activated receptor gamma (PPARγ), and alleviate neuroinflammation and ferroptosis in preclinical AD models.^14^^,^^45^^,^^137^^,^^143^^,^^219^ Pioglitazone reduces cognitive impairment by preventing lipid peroxidation.^220^ Thiazolidinediones ameliorate cerebral ischemia-reperfusion injury by combating overexpressed ACSL4 and decreasing ferroptosis.^16^ Intravenous rosiglitazone, administered before the middle cerebral artery occlusion in mice, significantly inhibited ACSL4 expression, increased GPX4 production, improved neurological function, and reduced infarct volume at 72 h post-stroke.^221^

AS-252424, a furan-2-ylmethylene thiazolidinedione, exhibits remarkable anti-ferroptosis activity by directly binding to glutamine 464 on ACSL4 and inhibiting its enzymatic function.^222^ Treatment with AS-252424-loaded nanoparticles effectively alleviates ferroptosis-mediated organ injury in multiple mouse models, including kidney ischemia-reperfusion injury and acute liver injury.^222^

MitoQ, a mitochondrial ROS scavenger with antioxidant properties, was discovered to prevent ferroptosis in HEK293T cells overexpressing ACSL4 and LPCAT2, highlighting the critical role that mitochondria play in ACSL4/LPCAT2-driven ferroptosis. In contrast, mitochondrial-energetic metabolic therapies fail to prevent ACSL4-mediated ferroptosis.^45^ Mitochondrial ROS production and the accompanying organelle disintegration are essential for mediating oxidative cell death initiated through lipid peroxidation in ferroptosis.^45^

Fer-1, a classic ferroptosis inhibitor, neutralizes hydrogen peroxide and lipid radicals.^180^^,^^223^ It reverses these effects that ACSL4 overexpression prevents macrophage M1 polarization from moving toward M2 polarization.^29^ Additionally, it can raise GSH levels, as well as decrease MDA and lipid peroxidation levels.^29^^,^^224^ Recent studies demonstrate that Fer-1 has a potent antioxidant capacity to inhibit ferroptosis in the brain, heart, kidney, and liver ischemia-reperfusion injury.^225^, ^226^, ^227^, ^228^

Edaravone, a lipophilic radical scavenger, eliminates LPOs and reduces oxidative stress, partly by inhibiting ACSL4 activation.^229^^,^^230^ In spinal cord injury models, edaravone up-regulates GPX4/xCT and down-regulates 5-lipoxygenase (5-LOX) and ACSL4, thereby promoting recovery via modulation of the GPX4/ACSL4/5-LOX pathway.^230^

### ACSL4 agonists

Thrombin, a serine protease, initiates ferroptosis signaling by encouraging AA mobilization and subsequent esterification by ACSL4.^231^ Tuo et al found that an ACSL4 inhibitor could counteract the cytotoxicity effects of thrombin, suggesting that thrombin may promote neuronal death through ACSL4-dependent ferroptosis.^231^ Besides, thrombin induces ferroptosis in triple-negative breast cancer through the cPLA2α/ACSL4 signaling pathway.^232^

Erastin, a classical ferroptosis inducer, regulates lipid peroxidation via controlling ACSL4. In glioblastoma cells, erastin enhances ACSL4 expression, which is positively regulated and stabilized by Hsp90 and dynamin-related protein 1 (Drp1).^233^ Activation of the Hsp90–ACSL4 axis enhances the anti-cancer efficacy of erastin both *in vitro* and *in vivo*.^233^

Glycochenodeoxycholic acid (GCDCA), a bile acid metabolite, induces ferroptosis by modulating the TFR–ACSL4 axis. This process contributes to inflammation and lipid metabolic disturbances following environmental toxin exposure.^234^

## Limitations and future perspectives

Targeting the ACSL4 pathway, while observing real and encouraging outcomes in various AD models and *in vitro* studies, has limitations that warrant consideration. Firstly, the mice mimic specific AD types with a defined genetic background, whereas the etiology of AD patients is often caused by complex genetic and genotype–environment interactions. For instance, ACSL4 is significantly down-regulated in the hippocampus of AD patients but up-regulated in APP/PS1 mice, highlighting inconsistencies likely due to species differences and the complexity of gene–environment interactions in human AD.^25^^,^^235^ Nowadays, almost all studies are still in the laboratory stage, and future studies must explore the role of ACSL4 in AD through clinical trials. The cooperation between basic and clinical research will elevate AD treatment to the next level. Secondly, while ACSL4 modulators show therapeutic potential, their safety profiles remain a major barrier to clinical application. Inhibiting ACSL4 enzymatic activity may interfere with physiological lipid metabolism. For instance, a novel ACSL4 inhibitor, PGRL493, inhibits *de novo* steroid synthesis in testicular and adrenal cells, which can disrupt hormone signaling and metabolism.^42^ Hence, it may be necessary to reduce drug side effects by optimizing drug carriers and improving drug targeting. Moreover, technologies enabling drug delivery across the blood-brain barrier create therapeutic platforms for AD.^236^^,^^237^ Prodrug strategies for AD, including chemically modifying drugs to improve their ability to penetrate the blood-brain-barrier and developing nanotechnology-based drug delivery systems to effectively penetrate the blood-brain-barrier, avoid cytotoxicity, and deliver to the brain, provide new ideas for the treatment of AD.^222^

Mounting evidence has suggested unique roles of ACSL4 in the field of neurodegenerative and tumor diseases.^16^^,^^29^^,^^233^ In fact, relatively little is known about the pathways related to targeting ACSL4 in AD-associated evidence and research. Moreover, whether the biological process of ACSL4 involved in AD is consistent with its participation in the control of tumors or other neurological conditions still needs convincing and more extensive verification. On the other hand, in the future, it will be necessary to explore that ACSL4 produced in various types of central nervous system cells, including astrocytes, neurons, microglia, oligodendrocytes, and vascular cells, has diverse roles in the AD pathogenesis course. Previous studies have confirmed that ACSL4 promotes neuronal ferroptosis and microglia-mediated neuroinflammation in ischemic stroke.^16^ This suggests that the molecular regulatory mechanisms involved in targeting ACSL4 in neurons and non-neurons are different, which may be caused by the inconsistent sensitivity of different cells to ferroptosis, or may be related to the different abundance of ACSL4 expression in different cell types in the brain. In addition, it is also worth studying the role of ACSL4 in immune cells. Indeed, ACSL4-mediated ferroptosis was identified earlier in the field of cancer. ACSL4 has been reported to modulate CD8^+^ T cell functions and impair anti-tumor immunity.^238^^,^^239^ Similarly, it has been demonstrated that ACSL4 and other ferroptosis-related molecules are dysregulated in AD patients, which can affect the infiltration of specific immune cell types.^25^ However, the mechanisms underlying ACSL4 for immune cell infiltration in the AD remain unclear until now. Given this complicated picture, it is critical to dissect the detrimental effects of ACSL4 at a cell type-specific level and to identify the underlying molecular mechanisms that drive AD pathogenesis in the most relevant cell populations.

## Concluding remarks

Advances in ferroptosis research have highlighted the pivotal role of ACSL4 in AD. The regulation of epigenetics and posttranslational modifications on the ACSL4 contributes to exploring the specific regulatory pathways of ACSL4 in AD in the future. Experimental evidence from animal and cellular models has shown that down-regulation of ACSL4 expression is of great significance in improving AD pathology and cognitive deficits. ACSL4, as the metabolic gatekeeper, plays an important role in the precise regulation of ferroptosis susceptibility, oxidative damage, and inflammatory response in AD. On the one hand, it regulates lipid remodeling to enhance ferroptosis susceptibility. On the other hand, ACSL4 is involved in oxidative stress by increasing ROS production and cytotoxic metabolite accumulation, as well as neuroinflammation by regulating microglial lipid metabolism and NF-κB signaling to enhance pro-inflammatory factor release. Therefore, inhibiting ACSL4 to enhance the defense against the aforesaid sequence of pathogenic mechanisms has a beneficial effect on AD treatment.

Certain synthetic and natural substances that function as ACSL4 inhibitors have demonstrated great clinical translational potential and have considerably enhanced cognitive abilities in preclinical studies. Despite the potential of novel compounds targeting to inhibit ACSL4 for clinical trials, some fundamental issues still need to be addressed beforehand. For example, Fer-1 inhibits ACSL4 expression in several experiments but lacks specificity.^29^ Thiazolidinediones effectively reduce the risk of AD in patients with diabetes or insulin resistance. However, low-dose pioglitazone failed to show efficacy in non-diabetic AD patients.^137^ AS-252424 attenuates lipid peroxidation and ferroptosis both *in vitro* and *in vivo*, but suffers from poor solubility, high clearance, and short half-life.^222^ PRGL493 inhibits AA-CoA synthesis and suppresses tumor growth in peripheral models but has not been studied in the central nervous system.^42^ While pharmacological inhibition of ACSL4 holds therapeutic potential in AD, limited information is available regarding the pharmacodynamics, pharmacokinetics, specificity, and efficacy of these reagents. More intensive efforts are obligatory to identify and develop brain-penetrant, specific, potent, and safe modulators of ACSL4. Future efforts should focus on elucidating ACSL4's exact molecular mechanisms and validating candidate compounds in robust preclinical models to facilitate clinical translation.

## CRediT authorship contribution statement

**Yu Guo:** Writing – original draft, Investigation, Conceptualization. **Qingqing Jiang:** Writing – review & editing, Supervision. **Zhongya Gu:** Writing – review & editing, Supervision. **Huan Cao:** Visualization. **Chengchao Zuo:** Writing – review & editing. **Yaqi Huang:** Writing – review & editing. **Yu Song:** Writing – review & editing. **Xiang Chen:** Writing – review & editing. **Furong Wang:** Writing – review & editing, Supervision, Conceptualization.

## Funding

This work was funded by the National Key Research and Development Program of Hubei Province (No. 2020BCA089) and the 10.13039/501100001809National Natural Science Foundation of China (No. 81974218).

## Conflict of interests

The authors have no relevant financial or nonfinancial interests to disclose.

## References

[1] Scheltens P., De Strooper B., Kivipelto M. (2021). Alzheimer's disease. Lancet.

[2] Wimo A., Seeher K., Cataldi R. (2023). The worldwide costs of dementia in 2019. Alzheimer's Dement.

[3] Li T., Lu L., Pember E., Li X., Zhang B., Zhu Z. (2022). New insights into neuroinflammation involved in pathogenic mechanism of Alzheimer's disease and its potential for therapeutic intervention. Cells.

[4] Benarroch E.E. (2009). Brain iron homeostasis and neurodegenerative disease. Neurology.

[5] Bartzokis G., Sultzer D., Mintz J. (1994). *In vivo* evaluation of brain iron in Alzheimer's disease and normal subjects using MRI. Biol Psychiatry.

[6] Ashraf A., Jeandriens J., Parkes H.G., So P.W. (2020). Iron dyshomeostasis, lipid peroxidation and perturbed expression of cystine/glutamate antiporter in Alzheimer's disease: evidence of ferroptosis. Redox Biol.

[7] Pietrzak R.H., Lim Y.Y., Ames D. (2015). Trajectories of memory decline in preclinical Alzheimer's disease: results from the Australian Imaging, Biomarkers and Lifestyle Flagship Study of ageing. Neurobiol Aging.

[8] Lemere C.A., Masliah E. (2010). Can Alzheimer disease be prevented by amyloid-beta immunotherapy?. Nat Rev Neurol.

[9] Ayton S., Lei P., Bush A.I. (2015). Biometals and their therapeutic implications in Alzheimer's disease. Neurotherapeutics.

[10] Ayton S., Faux N.G., Bush A.I. (2015). Ferritin levels in the cerebrospinal fluid predict Alzheimer's disease outcomes and are regulated by APOE. Nat Commun.

[11] Ayton S., Faux N.G., Bush A.I. (2017). Association of cerebrospinal fluid ferritin level with preclinical cognitive decline in APOE-ε4 carriers. JAMA Neurol.

[12] Kagan V.E., Mao G., Qu F. (2017). Oxidized arachidonic and adrenic PEs navigate cells to ferroptosis. Nat Chem Biol.

[13] Killion E.A., Reeves A.R., El Azzouny M.A. (2018). A role for long-chain acyl-CoA synthetase-4 (ACSL4) in diet-induced phospholipid remodeling and obesity-associated adipocyte dysfunction. Mol Metabol.

[14] Doll S., Proneth B., Tyurina Y.Y. (2017). ACSL4 dictates ferroptosis sensitivity by shaping cellular lipid composition. Nat Chem Biol.

[15] Dixon S.J., Winter G.E., Musavi L.S. (2015). Human haploid cell genetics reveals roles for lipid metabolism genes in nonapoptotic cell death. ACS Chem Biol.

[16] Cui Y., Zhang Y., Zhao X. (2021). ACSL4 exacerbates ischemic stroke by promoting ferroptosis-induced brain injury and neuroinflammation. Brain Behav Immun.

[17] Zhou X., Zhao R., Lv M. (2023). ACSL4 promotes microglia-mediated neuroinflammation by regulating lipid metabolism and VGLL4 expression. Brain Behav Immun.

[18] Dang D., Zhang C., Meng Z. (2022). Integrative analysis links ferroptosis to necrotizing enterocolitis and reveals the role of ACSL4 in immune disorders. iScience.

[19] Luoqian J., Yang W., Ding X. (2022). Ferroptosis promotes T-cell activation-induced neurodegeneration in multiple sclerosis. Cell Mol Immunol.

[20] Pei Z., Liu Y., Liu S. (2021). FUNDC1 insufficiency sensitizes high fat diet intake-induced cardiac remodeling and contractile anomaly through ACSL4-mediated ferroptosis. Metabolism.

[21] Sha R., Xu Y., Yuan C. (2021). Predictive and prognostic impact of ferroptosis-related genes *ACSL4* and GPX4 on breast cancer treated with neoadjuvant chemotherapy. EBioMedicine.

[22] Wang Y., Zhang M., Bi R. (2022). ACSL4 deficiency confers protection against ferroptosis-mediated acute kidney injury. Redox Biol.

[23] Zha X., Liu X., Wei M. (2025). Microbiota-derived lysophosphatidylcholine alleviates Alzheimer's disease pathology via suppressing ferroptosis. Cell Metab.

[24] Yang S., Wang L., Zeng Y. (2023). Salidroside alleviates cognitive impairment by inhibiting ferroptosis via activation of the Nrf2/GPX4 axis in SAMP8 mice. Phytomedicine.

[25] Zhang L., Fang J., Tang Z., Luo Y. (2022). A bioinformatics perspective on the dysregulation of ferroptosis and ferroptosis-related immune cell infiltration in Alzheimer's disease. Int J Med Sci.

[26] Zhu Z.Y., Liu Y.D., Gong Y. (2022). Mitochondrial aldehyde dehydrogenase (ALDH2) rescues cardiac contractile dysfunction in an APP/PS1 murine model of Alzheimer's disease via inhibition of ACSL4-dependent ferroptosis. Acta Pharmacol Sin.

[27] Zhang H., Zhou W., Li J. (2022). Senegenin rescues PC12 cells with oxidative damage through inhibition of ferroptosis. Mol Neurobiol.

[28] Wongjaikam S., Siengdee P., Somnus A., Govitrapong P. (2025). Melatonin alleviates erastin-induced cell death by inhibiting ferroptosis and amyloid precursor protein processing in neuronal cell lines. Neurotox Res.

[29] Chen P., Wang D., Xiao T. (2023). ACSL4 promotes ferroptosis and M1 macrophage polarization to regulate the tumorigenesis of nasopharyngeal carcinoma. Int Immunopharmacol.

[30] Zhang T.C., Lin Y.C., Sun N.N. (2024). Icariin, astragaloside a and puerarin mixture attenuates cognitive impairment in APP/PS1 mice via inhibition of ferroptosis-lipid peroxidation. Neurochem Int.

[31] Yuan Q., Wang M., Zhang Z. (2025). The ameliorative effects of melatonin against BDE-47-induced hippocampal neuronal ferroptosis and cognitive dysfunction through Nrf2-Chaperone-mediated autophagy of ACSL4 degradation. Ecotoxicol Environ Saf.

[32] Jiao H., Yang H., Yan Z. (2021). Traditional Chinese formula Xiaoyaosan alleviates depressive-like behavior in CUMS mice by regulating PEBP1-GPX4-mediated ferroptosis in the hippocampus. Neuropsychiatric Dis Treat.

[33] Kang M.J., Fujino T., Sasano H. (1997). A novel arachidonate-preferring acyl-CoA synthetase is present in steroidogenic cells of the rat adrenal, ovary, and testis. Proc Natl Acad Sci USA.

[34] Küch E.M., Vellaramkalayil R., Zhang I. (2014). Differentially localized acyl-CoA synthetase 4 isoenzymes mediate the metabolic channeling of fatty acids towards phosphatidylinositol. Biochim Biophys Acta BBA Mol Cell Biol Lipds.

[35] Quan J., Bode A.M., Luo X. (2021). ACSL family: the regulatory mechanisms and therapeutic implications in cancer. Eur J Pharmacol.

[36] Kassan A., Herms A., Fernández-Vidal A. (2013). Acyl-CoA synthetase 3 promotes lipid droplet biogenesis in ER microdomains. J Cell Biol.

[37] Jia B., Li J., Song Y., Luo C. (2023). ACSL4-mediated ferroptosis and its potential role in central nervous system diseases and injuries. Int J Mol Sci.

[38] Maloberti P., Lozano R.C., Mele P.G. (2002). Concerted regulation of free arachidonic acid and hormone-induced steroid synthesis by acyl-CoA thioesterases and acyl-CoA synthetases in adrenal cells. Eur J Biochem.

[39] Castilla R., Maloberti P., Castillo F. (2004). Arachidonic acid regulation of steroid synthesis: new partners in the signaling pathway of steroidogenic hormones. Endocr Res.

[40] Maloberti P., Castilla R., Castillo F. (2005). Silencing the expression of mitochondrial acyl-CoA thioesterase I and acyl-CoA synthetase 4 inhibits hormone-induced steroidogenesis. FEBS J.

[41] Maloberti P., Cornejo Maciel F., Castillo A.F. (2007). Enzymes involved in arachidonic acid release in adrenal and Leydig cells. Mol Cell Endocrinol.

[42] Castillo A.F., Orlando U.D., Maloberti P.M. (2021). New inhibitor targeting Acyl-CoA synthetase 4 reduces breast and prostate tumor growth, therapeutic resistance and steroidogenesis. Cell Mol Life Sci.

[43] Cornejo Maciel F., Maloberti P., Neuman I. (2005). An arachidonic acid-preferring acyl-CoA synthetase is a hormone-dependent and obligatory protein in the signal transduction pathway of steroidogenic hormones. J Mol Endocrinol.

[44] Paz C., Cornejo Maciel F., Gorostizaga A. (2016). Role of protein phosphorylation and tyrosine phosphatases in the adrenal regulation of steroid synthesis and mitochondrial function. Front Endocrinol.

[45] Merkel M., Goebel B., Boll M. (2023). Mitochondrial reactive oxygen species formation determines ACSL4/LPCAT2-mediated ferroptosis. Antioxidants.

[46] Golej D.L., Askari B., Kramer F. (2011). Long-chain acyl-CoA synthetase 4 modulates prostaglandin E_2_ release from human arterial smooth muscle cells. J Lipid Res.

[47] Liu Z., Huang Y., Zhang Y., Chen D., Zhang Y.Q. (2011). *Drosophila* Acyl-CoA synthetase long-chain family member 4 regulates axonal transport of synaptic vesicles and is required for synaptic development and transmission. J Neurosci.

[48] Yuan H., Li X., Zhang X., Kang R., Tang D. (2016). Identification of ACSL4 as a biomarker and contributor of ferroptosis. Biochem Biophys Res Commun.

[49] Miyares R.L., Stein C., Renisch B., Anderson J.L., Hammerschmidt M., Farber S.A. (2013). Long-chain Acyl-CoA synthetase 4A regulates Smad activity and dorsoventral patterning in the zebrafish embryo. Dev Cell.

[50] Nakahara K., Ohkuni A., Kitamura T. (2012). The Sjögren-Larsson syndrome gene encodes a hexadecenal dehydrogenase of the sphingosine 1-phosphate degradation pathway. Mol Cell.

[51] Chen J., Fu C.Y., Shen G. (2022). Macrophages induce cardiomyocyte ferroptosis via mitochondrial transfer. Free Radic Biol Med.

[52] Tang Y., Zhou J., Hooi S.C., Jiang Y.M., Lu G.D. (2018). Fatty acid activation in carcinogenesis and cancer development: essential roles of long-chain acyl-CoA synthetases. Oncol Lett.

[53] Soupene E., Kuypers F.A. (2008). Mammalian long-chain acyl-CoA synthetases. Exp Biol Med (Maywood).

[54] Lin J., Lai Y., Lu F., Wang W. (2025). Targeting ACSLs to modulate ferroptosis and cancer immunity. Trends Endocrinol Metab.

[55] Singh A.B., Dong B., Xu Y., Zhang Y., Liu J. (2019). Identification of a novel function of hepatic long-chain acyl-CoA synthetase-1 (ACSL1) in bile acid synthesis and its regulation by bile acid-activated farnesoid X receptor. Biochim Biophys Acta Mol Cell Biol Lipids.

[56] Tsushima K., Bugger H., Wende A.R. (2018). Mitochondrial reactive oxygen species in lipotoxic hearts induce post-translational modifications of AKAP121, DRP1, and OPA1 that promote mitochondrial fission. Circ Res.

[57] Zhao L., Pascual F., Bacudio L. (2019). Defective fatty acid oxidation in mice with muscle-specific acyl-CoA synthetase 1 deficiency increases amino acid use and impairs muscle function. J Biol Chem.

[58] Ellis J.M., Li L.O., Wu P.C. (2010). Adipose acyl-CoA synthetase-1 directs fatty acids toward beta-oxidation and is required for cold thermogenesis. Cell Metab.

[59] Lobo S., Wiczer B.M., Bernlohr D.A. (2009). Functional analysis of long-chain acyl-CoA synthetase 1 in 3T3-L1 adipocytes. J Biol Chem.

[60] Huh J.Y., Reilly S.M., Abu-Odeh M. (2020). TANK-binding kinase 1 regulates the localization of acyl-CoA synthetase ACSL1 to control hepatic fatty acid oxidation. Cell Metab.

[61] Zhang L., Wu B., Wang D. (2023). The mechanism of long-chain acyl-CoA synthetase 3 in inhibiting cell proliferation, migration, and invasion in clear cell renal cell carcinoma. Am J Cancer Res.

[62] Magtanong L., Ko P.J., To M. (2019). Exogenous monounsaturated fatty acids promote a ferroptosis-resistant cell state. Cell Chem Biol.

[63] Klaus C., Kaemmerer E., Reinartz A. (2014). TP53 status regulates ACSL5-induced expression of mitochondrial mortalin in enterocytes and colorectal adenocarcinomas. Cell Tissue Res.

[64] Gassler N., Roth W., Funke B. (2007). Regulation of enterocyte apoptosis by acyl-CoA synthetase 5 splicing. Gastroenterology.

[65] Bowman T.A., O'Keeffe K.R., D'Aquila T. (2016). Acyl CoA synthetase 5 (ACSL5) ablation in mice increases energy expenditure and insulin sensitivity and delays fat absorption. Mol Metabol.

[66] Grevengoed T.J., Klett E.L., Coleman R.A. (2014). Acyl-CoA metabolism and partitioning. Annu Rev Nutr.

[67] Mashek D.G., McKenzie M.A., Van Horn C.G., Coleman R.A. (2006). Rat long chain acyl-CoA synthetase 5 increases fatty acid uptake and partitioning to cellular triacylglycerol in McArdle-RH7777 cells. J Biol Chem.

[68] Fernandez R.F., Kim S.Q., Zhao Y. (2018). Acyl-CoA synthetase 6 enriches the neuroprotective omega-3 fatty acid DHA in the brain. Proc Natl Acad Sci USA.

[69] Durgan D.J., Smith J.K., Hotze M.A. (2006). Distinct transcriptional regulation of long-chain acyl-CoA synthetase isoforms and cytosolic thioesterase 1 in the rodent heart by fatty acids and insulin. Am J Physiol Heart Circ Physiol.

[70] Basil M.C., Levy B.D. (2016). Specialized pro-resolving mediators: endogenous regulators of infection and inflammation. Nat Rev Immunol.

[71] Dennis E.A., Norris P.C. (2015). Eicosanoid storm in infection and inflammation. Nat Rev Immunol.

[72] Serhan C.N. (2014). Pro-resolving lipid mediators are leads for resolution physiology. Nature.

[73] Hong S., Gronert K., Devchand P.R., Moussignac R.L., Serhan C.N. (2003). Novel docosatrienes and 17S-resolvins generated from docosahexaenoic acid in murine brain, human blood, and glial cells. Autacoids in anti-inflammation. J Biol Chem.

[74] Tang D., Chen X., Kang R., Kroemer G. (2021). Ferroptosis: molecular mechanisms and health implications. Cell Res.

[75] Chen H., Ren L., Ma W. (2023). Mechanism of SOX10 in ferroptosis of hippocampal neurons after intracerebral hemorrhage via the miR-29a-3p/ACSL4 axis. J Neurophysiol.

[76] Feng S., Rao Z., Zhang J. (2023). Inhibition of CARM1-mediated methylation of ACSL4 promotes ferroptosis in colorectal cancer. Adv Sci (Weinh).

[77] Chen C., Yang Y., Guo Y. (2023). CYP1B1 inhibits ferroptosis and induces anti-PD-1 resistance by degrading ACSL4 in colorectal cancer. Cell Death Dis.

[78] Cavalli G., Heard E. (2019). Advances in epigenetics link genetics to the environment and disease. Nature.

[79] Cao J., Yan Q. (2020). Cancer epigenetics, tumor immunity, and immunotherapy. Trends Cancer.

[80] Wang N., Ma T., Yu B. (2023). Targeting epigenetic regulators to overcome drug resistance in cancers. Signal Transduct Targeted Ther.

[81] Wu X., Xu M., Geng M. (2023). Targeting protein modifications in metabolic diseases: molecular mechanisms and targeted therapies. Signal Transduct Targeted Ther.

[82] Zhong Q., Xiao X., Qiu Y. (2023). Protein posttranslational modifications in health and diseases: functions, regulatory mechanisms, and therapeutic implications. MedComm.

[83] Pezzi J.C., Ens C.M.B., Borba E.M. (2014). DNA methyltransferase haplotype is associated with Alzheimer's disease. Neurosci Lett.

[84] Jaenisch R., Bird A. (2003). Epigenetic regulation of gene expression: how the genome integrates intrinsic and environmental signals. Nat Genet.

[85] Rutten B.P.F., Mill J. (2009). Epigenetic mediation of environmental influences in major psychotic disorders. Schizophr Bull.

[86] Chen T., Li E. (2006). Establishment and maintenance of DNA methylation patterns in mammals. Curr Top Microbiol Immunol.

[87] Agarwal S., Amin K.S., Jagadeesh S. (2013). Mahanine restores RASSF1A expression by down-regulating DNMT1 and DNMT3B in prostate cancer cells. Mol Cancer.

[88] Bao Q., He B., Pan Y. (2011). Genetic variation in the promoter of DNMT3B is associated with the risk of colorectal cancer. Int J Colorectal Dis.

[89] Haggarty P., Hoad G., Harris S.E. (2010). Human intelligence and polymorphisms in the DNA methyltransferase genes involved in epigenetic marking. PLoS One.

[90] Higuchi F., Uchida S., Yamagata H. (2011). State-dependent changes in the expression of DNA methyltransferases in mood disorder patients. J Psychiatr Res.

[91] Zhang C., Fang Y., Xie B. (2009). DNA methyltransferase 3B gene increases risk of early onset schizophrenia. Neurosci Lett.

[92] Tong Z., Han C., Qiang M. (2015). Age-related formaldehyde interferes with DNA methyltransferase function, causing memory loss in Alzheimer's disease. Neurobiol Aging.

[93] Yankova E., Blackaby W., Albertella M. (2021). Small-molecule inhibition of METTL3 as a strategy against myeloid leukaemia. Nature.

[94] Wang W., Chen J., Lai S. (2025). METTL14 promotes ferroptosis in smooth muscle cells during thoracic aortic aneurysm by stabilizing the m^6^A modification of ACSL4. Am J Physiol Cell Physiol.

[95] Wu J., Minikes A.M., Gao M. (2019). Intercellular interaction dictates cancer cell ferroptosis via NF2-YAP signalling. Nature.

[96] Chen B., Wang H., Lv C., Mao C., Cui Y. (2021). Long non-coding RNA H19 protects against intracerebral hemorrhage injuries via regulating microRNA-106b-5p/acyl-CoA synthetase long chain family member 4 axis. Bioengineered.

[97] Lu Y., Chan Y.T., Tan H.Y. (2022). Epigenetic regulation of ferroptosis via ETS1/miR-23a-3p/ACSL4 axis mediates sorafenib resistance in human hepatocellular carcinoma. J Exp Clin Cancer Res.

[98] Esteller M. (2011). Non-coding RNAs in human disease. Nat Rev Genet.

[99] Wei J.W., Huang K., Yang C. (2017). Non-coding RNAs as regulators in epigenetics. Oncol Rep.

[100] Kou X., Chen D., Chen N. (2020). The regulation of microRNAs in Alzheimer's disease. Front Neurol.

[101] Li W., Shan B.Q., Zhao H.Y. (2022). miR-130a-3p regulates neural stem cell differentiation *in vitro* by targeting Acsl4. J Cell Mol Med.

[102] Mao R., Liu H. (2022). Depletion of mmu_circ_0001751 (circular RNA Carm1) protects against acute cerebral infarction injuries by binding with microRNA-3098-3p to regulate acyl-CoA synthetase long-chain family member 4. Bioengineered.

[103] Zhang M., Yu Z., Zhao L., Luo H. (2023). Long non-coding RNA PVT1 regulates atherosclerosis progression via the microRNA-106b-5p/ACSL4 axis. Biochem Biophys Res Commun.

[104] Lei G., Horbath A., Li Z., Gan B. (2022). PKCβII-ACSL4 pathway mediating ferroptosis execution and anti-tumor immunity. Cancer Commun (Lond).

[105] Zhang H.L., Hu B.X., Li Z.L. (2022). PKCβII phosphorylates ACSL4 to amplify lipid peroxidation to induce ferroptosis. Nat Cell Biol.

[106] Li Z., Xu Z.M., Chen W.P. (2024). Tumor-repopulating cells evade ferroptosis via PCK2-dependent phospholipid remodeling. Nat Chem Biol.

[107] Zeng K., Li W., Wang Y. (2023). Inhibition of CDK1 overcomes oxaliplatin resistance by regulating ACSL4-mediated ferroptosis in colorectal cancer. Adv Sci (Weinh).

[108] Xie G., Li N., Li K. (2024). Phosphatase LHPP confers prostate cancer ferroptosis activation by modulating the AKT-SKP2-ACSL4 pathway. Cell Death Dis.

[109] Cockram P.E., Kist M., Prakash S., Chen S.H., Wertz I.E., Vucic D. (2021). Ubiquitination in the regulation of inflammatory cell death and cancer. Cell Death Differ.

[110] Zhuo B., Qin C., Deng S. (2025). The role of ACSL4 in stroke: mechanisms and potential therapeutic target. Mol Cell Biochem.

[111] Bao Z., Liu Y., Chen B. (2021). Prokineticin-2 prevents neuronal cell deaths in a model of traumatic brain injury. Nat Commun.

[112] Sun Y., Jin H., He J., Lai J., Lin H., Liu X. (2024). Melatonin alleviates ischemic stroke by inhibiting ferroptosis through the CYP1B1/ACSL4 pathway. Environ Toxicol.

[113] Qiu M., Yan W., Liu M. (2023). YAP facilitates NEDD4L-mediated ubiquitination and degradation of ACSL4 to alleviate ferroptosis in myocardial ischemia-reperfusion injury. Can J Cardiol.

[114] Xiao D., Chang W., Ao X. (2025). Parkin inhibits iron overload-induced cardiomyocyte ferroptosis by ubiquitinating ACSL4 and modulating PUFA-phospholipids metabolism. Acta Pharm Sin B.

[115] Huang Y., Wang S., Hu D., Zhang L., Shi S. (2025). Inhibition of LPL suppresses the osteoclast differentiation of bone-marrow-derived macrophages by promoting the ACSL4 ubiquitination. Int Immunopharmacol.

[116] Nguyen K.T., Mun S.H., Yang J. (2022). The MARCHF6 E3 ubiquitin ligase acts as an NADPH sensor for the regulation of ferroptosis. Nat Cell Biol.

[117] Yang X., Zhang Z., Wang F. (2025). Capsaicin inhibits ferroptosis through facilitating SYVN1-mediated ubiquitination and degradation of ACSL4. J Agric Food Chem.

[118] Zhao X. (2018). SUMO-mediated regulation of nuclear functions and signaling processes. Mol Cell.

[119] Chang H.M., Yeh E.T.H. (2020). SUMO: from bench to bedside. Physiol Rev.

[120] Gareau J.R., Lima C.D. (2010). The SUMO pathway: emerging mechanisms that shape specificity, conjugation and recognition. Nat Rev Mol Cell Biol.

[121] Hickey C.M., Wilson N.R., Hochstrasser M. (2012). Function and regulation of SUMO proteases. Nat Rev Mol Cell Biol.

[122] Salas-Lloret D., González-Prieto R. (2022). Insights in post-translational modifications: ubiquitin and SUMO. Int J Mol Sci.

[123] Liu W., Zhu Y., Ye W. (2025). Redox regulation of TRIM28 facilitates neuronal ferroptosis by promoting SUMOylation and inhibiting OPTN-selective autophagic degradation of ACSL4. Cell Death Differ.

[124] Xu X., Mao Y., Feng Z., Dai F., Gu T., Zheng J. (2024). SENP1 inhibits ferroptosis and promotes head and neck squamous cell carcinoma by regulating ACSL4 protein stability via SUMO1. Oncol Rep.

[125] Fioriti L., Wijesekara N., Argyrousi E.K. (2025). Genetic and pharmacologic enhancement of SUMO2 conjugation prevents and reverses cognitive impairment and synaptotoxicity in a preclinical model of Alzheimer's disease. Alzheimer's Dement.

[126] Saha R.N., Pahan K. (2006). HATs and HDACs in neurodegeneration: a tale of disconcerted acetylation homeostasis. Cell Death Differ.

[127] Zhou P., Peng X., Zhang K. (2025). HAT1/HDAC2 mediated ACSL4 acetylation confers radiosensitivity by inducing ferroptosis in nasopharyngeal carcinoma. Cell Death Dis.

[128] Hou J., Jiang C., Wen X. (2022). ACSL4 as a potential target and biomarker for anticancer: from molecular mechanisms to clinical therapeutics. Front Pharmacol.

[129] Jiang L., Kon N., Li T. (2015). Ferroptosis as a p53-mediated activity during tumour suppression. Nature.

[130] Wang H., Liu C., Zhao Y., Gao G. (2020). Mitochondria regulation in ferroptosis. Eur J Cell Biol.

[131] Catalá A., Díaz M. (2016). Editorial: impact of lipid peroxidation on the physiology and pathophysiology of cell membranes. Front Physiol.

[132] Ashraf A., Clark M., So P.W. (2018). The aging of iron man. Front Aging Neurosci.

[133] Ward R.J., Zucca F.A., Duyn J.H., Crichton R.R., Zecca L. (2014). The role of iron in brain ageing and neurodegenerative disorders. Lancet Neurol.

[134] McAllum E.J., Hare D.J., Volitakis I. (2020). Regional iron distribution and soluble ferroprotein profiles in the healthy human brain. Prog Neurobiol.

[135] Raha A.A., Vaishnav R.A., Friedland R.P., Bomford A., Raha-Chowdhury R. (2013). The systemic iron-regulatory proteins hepcidin and ferroportin are reduced in the brain in Alzheimer's disease. Acta Neuropathol Commun.

[136] Barrera G., Pizzimenti S., Daga M. (2018). Lipid peroxidation-derived aldehydes, 4-hydroxynonenal and malondialdehyde in aging-related disorders. Antioxidants (Basel).

[137] Yang J., Shi X., Wang Y. (2023). Multi-target neuroprotection of thiazolidinediones on Alzheimer's disease via neuroinflammation and ferroptosis. J Alzheimers Dis.

[138] Fuhrmann D.C., Brüne B. (2022). A graphical journey through iron metabolism, microRNAs, and hypoxia in ferroptosis. Redox Biol.

[139] Doll S., Freitas F.P., Shah R. (2019). FSP1 is a glutathione-independent ferroptosis suppressor. Nature.

[140] Bersuker K., Hendricks J.M., Li Z. (2019). The CoQ oxidoreductase FSP1 acts parallel to GPX4 to inhibit ferroptosis. Nature.

[141] Yan H.F., Zou T., Tuo Q.Z. (2021). Ferroptosis: mechanisms and links with diseases. Signal Transduct Targeted Ther.

[142] Guo W., Zhang C., Zhou Q. (2025). Mitochondrial CCN1 drives ferroptosis via fatty acid β-oxidation. Dev Cell.

[143] Van Horn C.G., Caviglia J.M., Li L.O., Wang S., Granger D.A., Coleman R.A. (2005). Characterization of recombinant long-chain rat acyl-CoA synthetase isoforms 3 and 6: Identification of a novel variant of isoform 6. Biochemistry.

[144] Wang X., Li S., Yu J. (2023). Saikosaponin B2 ameliorates depression-induced microglia activation by inhibiting ferroptosis-mediated neuroinflammation and ER stress. J Ethnopharmacol.

[145] Butterfield D.A., Boyd-Kimball D. (2018). Oxidative stress, amyloid-β peptide, and altered key molecular pathways in the pathogenesis and progression of Alzheimer's disease. J Alzheimers Dis.

[146] Becerril-Ortega J., Bordji K., Fréret T., Rush T., Buisson A. (2014). Iron overload accelerates neuronal amyloid-β production and cognitive impairment in transgenic mice model of Alzheimer's disease. Neurobiol Aging.

[147] Kim A.C., Lim S., Kim Y.K. (2018). Metal ion effects on aβ and tau aggregation. Int J Mol Sci.

[148] James S.A., Churches Q.I., de Jonge M.D. (2017). Iron, copper, and zinc concentration in aβ plaques in the APP/PS1 mouse model of Alzheimer's disease correlates with metal levels in the surrounding neuropil. ACS Chem Neurosci.

[149] Spotorno N., Acosta-Cabronero J., Stomrud E. (2020). Relationship between cortical iron and tau aggregation in Alzheimer's disease. Brain.

[150] Guillemot J., Canuel M., Essalmani R., Prat A., Seidah N.G. (2013). Implication of the proprotein convertases in iron homeostasis: proprotein convertase 7 sheds human transferrin receptor 1 and furin activates hepcidin. Hepatology.

[151] Silvestri L., Camaschella C. (2008). A potential pathogenetic role of iron in Alzheimer's disease. J Cell Mol Med.

[152] Belaidi A.A., Bush A.I. (2016). Iron neurochemistry in Alzheimer's disease and Parkinson's disease: targets for therapeutics. J Neurochem.

[153] Hambright W.S., Fonseca R.S., Chen L., Na R., Ran Q. (2017). Ablation of ferroptosis regulator glutathione peroxidase 4 in forebrain neurons promotes cognitive impairment and neurodegeneration. Redox Biol.

[154] Chen L., Dar N.J., Na R. (2022). Enhanced defense against ferroptosis ameliorates cognitive impairment and reduces neurodegeneration in 5xFAD mice. Free Radic Biol Med.

[155] Greenough M.A., Lane D.J.R., Balez R. (2022). Selective ferroptosis vulnerability due to familial Alzheimer's disease presenilin mutations. Cell Death Differ.

[156] Sultana R., Perluigi M., Butterfield D.A. (2006). Protein oxidation and lipid peroxidation in brain of subjects with Alzheimer's disease: insights into mechanism of neurodegeneration from redox proteomics. Antioxidants Redox Signal.

[157] Chen C.T., Green J.T., Orr S.K., Bazinet R.P. (2008). Regulation of brain polyunsaturated fatty acid uptake and turnover. Prostagl Leukot Essent Fat Acids.

[158] Angelova P.R., Abramov A.Y. (2016). Functional role of mitochondrial reactive oxygen species in physiology. Free Radic Biol Med.

[159] Chen X., Li J., Kang R., Klionsky D.J., Tang D. (2021). Ferroptosis: machinery and regulation. Autophagy.

[160] Schaur R.J. (2003). Basic aspects of the biochemical reactivity of 4-hydroxynonenal. Mol Aspect Med.

[161] Esterbauer H., Schaur R.J., Zollner H. (1991). Chemistry and biochemistry of 4-hydroxynonenal, malonaldehyde and related aldehydes. Free Radic Biol Med.

[162] Shearn C.T., Fritz K.S., Shearn A.H. (2016). Deletion of GSTA4-4 results in increased mitochondrial post-translational modification of proteins by reactive aldehydes following chronic ethanol consumption in mice. Redox Biol.

[163] Williams T.I., Lynn B.C., Markesbery W.R., Lovell M.A. (2006). Increased levels of 4-hydroxynonenal and acrolein, neurotoxic markers of lipid peroxidation, in the brain in mild cognitive impairment and early Alzheimer's disease. Neurobiol Aging.

[164] Ansari M.A., Scheff S.W. (2010). Oxidative stress in the progression of Alzheimer disease in the frontal cortex. J Neuropathol Exp Neurol.

[165] Bradley M.A., Markesbery W.R., Lovell M.A. (2010). Increased levels of 4-hydroxynonenal and acrolein in the brain in preclinical Alzheimer disease. Free Radic Biol Med.

[166] Chen C., Lu J., Peng W. (2022). Acrolein, an endogenous aldehyde induces Alzheimer's disease-like pathologies in mice: a new sporadic AD animal model. Pharmacol Res.

[167] Montine K.S., Quinn J.F., Zhang J. (2004). Isoprostanes and related products of lipid peroxidation in neurodegenerative diseases. Chem Phys Lipids.

[168] Li C., Hua C., Chu C. (2025). A photothermal-responsive multi-enzyme nanoprobe for ROS amplification and glutathione depletion to enhance ferroptosis. Biosens Bioelectron.

[169] Shen Y., Zhang G., Wei C. (2025). Potential role and therapeutic implications of glutathione peroxidase 4 in the treatment of Alzheimer's disease. Neural Regen Res.

[170] Magtanong L., Mueller G.D., Williams K.J. (2022). Context-dependent regulation of ferroptosis sensitivity. Cell Chem Biol.

[171] Singhal G., Jaehne E.J., Corrigan F., Toben C., Baune B.T. (2014). Inflammasomes in neuroinflammation and changes in brain function: a focused review. Front Neurosci.

[172] Haney M.S., Pálovics R., Munson C.N. (2024). APOE4/4 is linked to damaging lipid droplets in Alzheimer's disease microglia. Nature.

[173] Kuwata H., Nakatani E., Shimbara-Matsubayashi S. (2019). Long-chain acyl-CoA synthetase 4 participates in the formation of highly unsaturated fatty acid-containing phospholipids in murine macrophages. Biochim Biophys Acta Mol Cell Biol Lipids.

[174] Reeves A.R., Sansbury B.E., Pan M., Han X., Spite M., Greenberg A.S. (2021). Myeloid-specific deficiency of long-chain acyl CoA synthetase 4 reduces inflammation by remodeling phospholipids and reducing production of arachidonic acid-derived proinflammatory lipid mediators. J Immunol.

[175] Lei G., Zhuang L., Gan B. (2024). The roles of ferroptosis in cancer: tumor suppression, tumor microenvironment, and therapeutic interventions. Cancer Cell.

[176] Sun Y., Chen P., Zhai B. (2020). The emerging role of ferroptosis in inflammation. Biomed Pharmacother.

[177] Kim E.H., Wong S.W., Martinez J. (2019). Programmed necrosis and disease: we interrupt your regular programming to bring you necroinflammation. Cell Death Differ.

[178] Latchoumycandane C., Marathe G.K., Zhang R., McIntyre T.M. (2012). Oxidatively truncated phospholipids are required agents of tumor necrosis factor α (TNFα)-induced apoptosis. J Biol Chem.

[179] Urrutia P.J., Bórquez D.A., Núñez M.T. (2021). Inflaming the brain with iron. Antioxidants (Basel).

[180] Kajarabille N., Latunde-Dada G.O. (2019). Programmed cell-death by ferroptosis: antioxidants as mitigators. Int J Mol Sci.

[181] Zhang J., Tan B., Wu H. (2025). *Scutellaria baicalensis* extracts restrict intestinal epithelial cell ferroptosis by regulating lipid peroxidation and GPX4/ACSL4 in colitis. Phytomedicine.

[182] Tian M., Shen J., Qi Z., Feng Y., Fang P. (2023). Bioinformatics analysis and prediction of Alzheimer's disease and alcohol dependence based on ferroptosis-related genes. Front Aging Neurosci.

[183] Stockwell B.R., Friedmann Angeli J.P., Bayir H. (2017). Ferroptosis: a regulated cell death nexus linking metabolism, redox biology, and disease. Cell.

[184] Bao C., Liu C., Liu Q. (2022). Liproxstatin-1 alleviates LPS/IL-13-induced bronchial epithelial cell injury and neutrophilic asthma in mice by inhibiting ferroptosis. Int Immunopharmacol.

[185] Tsurusaki S., Tsuchiya Y., Koumura T. (2019). Hepatic ferroptosis plays an important role as the trigger for initiating inflammation in nonalcoholic steatohepatitis. Cell Death Dis.

[186] Cao Y., Li Y., He C. (2021). Selective ferroptosis inhibitor liproxstatin-1 attenuates neurological deficits and neuroinflammation after subarachnoid hemorrhage. Neurosci Bull.

[187] Shou Y., Yang L., Yang Y., Xu J. (2021). Inhibition of keratinocyte ferroptosis suppresses psoriatic inflammation. Cell Death Dis.

[188] Maloberti P.M., Duarte A.B., Orlando U.D. (2010). Functional interaction between acyl-CoA synthetase 4, lipooxygenases and cyclooxygenase-2 in the aggressive phenotype of breast cancer cells. PLoS One.

[189] Smith W.L., Langenbach R. (2001). Why there are two cyclooxygenase isozymes. J Clin Investig.

[190] Brash A.R. (2001). Arachidonic acid as a bioactive molecule. J Clin Investig.

[191] Thomas M.H., Paris C., Magnien M. (2017). Dietary arachidonic acid increases deleterious effects of amyloid-β oligomers on learning abilities and expression of AMPA receptors: putative role of the ACSL4-cPLA(2) balance. Alzheimers Res Ther.

[192] Jilg A., Bechstein P., Saade A. (2019). Melatonin modulates daytime-dependent synaptic plasticity and learning efficiency. J Pineal Res.

[193] Wu Y.H., Swaab D.F. (2005). The human pineal gland and melatonin in aging and Alzheimer's disease. J Pineal Res.

[194] Gao T., Wang Z., Dong Y. (2019). Role of melatonin in sleep deprivation-induced intestinal barrier dysfunction in mice. J Pineal Res.

[195] Lv W.J., Liu C., Yu L.Z. (2020). Melatonin alleviates neuroinflammation and metabolic disorder in DSS-induced depression rats. Oxid Med Cell Longev.

[196] Leaden P.J., Catalá A. (2005). Protective effect of melatonin on ascorbate-Fe^2+^ lipid peroxidation of polyunsaturated fatty acids in rat liver, kidney and brain microsomes: a chemiluminescence study. J Pineal Res.

[197] Ji Q., Zhang L., Ye H. (2024). Melatonin improves stroke through MDM2-mediated ubiquitination of ACSL4. Aging.

[198] Chang Y.S., Tsai C.T., Huangfu C.A. (2011). ACSL3 and GSK-3β are essential for lipid upregulation induced by endoplasmic reticulum stress in liver cells. J Cell Biochem.

[199] Kaemmerer E., Peuscher A., Reinartz A. (2011). Human intestinal acyl-CoA synthetase 5 is sensitive to the inhibitor triacsin C. World J Gastroenterol.

[200] Yen M.C., Kan J.Y., Hsieh C.J., Kuo P.L., Hou M.F., Hsu Y.L. (2017). Association of long-chain acyl-coenzyme A synthetase 5 expression in human breast cancer by estrogen receptor status and its clinical significance. Oncol Rep.

[201] Xin W., Pan Y., Wei W. (2023). TGF-β1 decreases microglia-mediated neuroinflammation and lipid droplet accumulation in an *in vitro* stroke model. Int J Mol Sci.

[202] Odorcyk F.K., Duran-Carabali L.E., Rocha D.S. (2020). Differential glucose and beta-hydroxybutyrate metabolism confers an intrinsic neuroprotection to the immature brain in a rat model of neonatal hypoxia ischemia. Exp Neurol.

[203] Yu X., Yang Y., Zhang B. (2023). Ketone body β-hydroxybutyric acid ameliorates dopaminergic neuron injury through modulating zinc finger protein 36/acyl-CoA synthetase long-chain family member four signaling axis-mediated ferroptosis. Neuroscience.

[204] Lee B.S., Woo D.C., Woo C.W., Kim K.S. (2018). Exogenous β-hydroxybutyrate treatment and neuroprotection in a suckling rat model of hypoxic-ischemic encephalopathy. Dev Neurosci.

[205] Baral S., Pariyar R., Yoon C.S. (2015). Effects of Gastrodiae rhizoma on proliferation and differentiation of human embryonic neural stem cells. Asian Pac J Tropical Med.

[206] Hsu W.H., Huang N.K., Shiao Y.J. (2021). Gastrodiae rhizoma attenuates brain aging via promoting neuritogenesis and neurodifferentiation. Phytomedicine.

[207] Zhao W., Wang J., Latta M. (2022). Rhizoma gastrodiae water extract modulates the gut microbiota and pathological changes of P-tau(Thr231) to protect against cognitive impairment in mice. Front Pharmacol.

[208] Lou T., Wu H., Feng M. (2024). Integration of metabolomics and transcriptomics reveals that Da Chuanxiong Formula improves vascular cognitive impairment via ACSL4/GPX4 mediated ferroptosis. J Ethnopharmacol.

[209] Chen G., Chen X., Niu C. (2019). Baicalin alleviates hyperglycemia-induced endothelial impairment via Nrf2. J Endocrinol.

[210] Fang J., Wang H., Zhou J. (2018). Baicalin provides neuroprotection in traumatic brain injury mice model through Akt/Nrf2 pathway. Drug Des Dev Ther.

[211] Shi X., Fu Y., Zhang S., Ding H., Chen J. (2017). Baicalin attenuates subarachnoid hemorrhagic brain injury by modulating blood-brain barrier disruption, inflammation, and oxidative damage in mice. Oxid Med Cell Longev.

[212] Wang Z., Ma L., Su M. (2018). Baicalin induces cellular senescence in human colon cancer cells via upregulation of DEPP and the activation of Ras/Raf/MEK/ERK signaling. Cell Death Dis.

[213] Duan L., Zhang Y., Yang Y. (2021). Baicalin inhibits ferroptosis in intracerebral hemorrhage. Front Pharmacol.

[214] Wu X., Zhi F., Lun W., Deng Q., Zhang W. (2018). Baicalin inhibits PDGF-BB-induced hepatic stellate cell proliferation, apoptosis, invasion, migration and activation via the miR-3595/ACSL4 axis. Int J Mol Med.

[215] Cai M., Shao W., Yu H., Hong Y., Shi L. (2020). Paeonol inhibits cell proliferation, migration and invasion and induces apoptosis in hepatocellular carcinoma by regulating miR-21-5p/KLF6 axis. Cancer Manag Res.

[216] Shi X., Xie X., Sun Y. (2020). Paeonol inhibits NLRP3 mediated inflammation in rat endothelial cells by elevating hyperlipidemic rats plasma exosomal miRNA-223. Eur J Pharmacol.

[217] Yu Y., Yan R., Chen X., Sun T., Yan J. (2020). Paeonol suppresses the effect of ox-LDL on mice vascular endothelial cells by regulating miR-338-3p/TET2 axis in atherosclerosis. Mol Cell Biochem.

[218] Jin Z.L., Gao W.Y., Liao S.J. (2021). Paeonol inhibits the progression of intracerebral haemorrhage by mediating the HOTAIR/UPF1/ACSL4 axis. ASN Neuro.

[219] Kim J.H., Lewin T.M., Coleman R.A. (2001). Expression and characterization of recombinant rat Acyl-CoA synthetases 1, 4, and 5. Selective inhibition by triacsin C and thiazolidinediones. J Biol Chem.

[220] Alzoubi K.H., Khabour O.F., Alfaqih M. (2022). The protective effects of pioglitazone against cognitive impairment caused by L-methionine administration in a rat model. CNS Neurol Disord: Drug Targets.

[221] Chen J., Yang L., Geng L. (2021). Inhibition of acyl-CoA synthetase long-chain family member 4 facilitates neurological recovery after stroke by regulation ferroptosis. Front Cell Neurosci.

[222] Huang Q., Ru Y., Luo Y. (2024). Identification of a targeted ACSL4 inhibitor to treat ferroptosis-related diseases. Sci Adv.

[223] Dixon S.J., Lemberg K.M., Lamprecht M.R. (2012). Ferroptosis: an iron-dependent form of nonapoptotic cell death. Cell.

[224] Liu S., Chen F., Han J., Wang L., Dong Y. (2024). Ferrostatin-1 improves neurological impairment induced by ischemia/reperfusion injury in the spinal cord through ERK1/2/SP1/GPX4. Exp Neurol.

[225] Liu X., Du Y., Liu J., Cheng L., He W., Zhang W. (2023). Ferrostatin-1 alleviates cerebral ischemia/reperfusion injury through activation of the AKT/GSK3β signaling pathway. Brain Res Bull.

[226] Stamenkovic A., O'Hara K.A., Nelson D.C. (2021). Oxidized phosphatidylcholines trigger ferroptosis in cardiomyocytes during ischemia-reperfusion injury. Am J Physiol Heart Circ Physiol.

[227] Fan X., Zhang X., Liu L.C. (2022). Hemopexin accumulates in kidneys and worsens acute kidney injury by causing hemoglobin deposition and exacerbation of iron toxicity in proximal tubules. Kidney Int.

[228] Yamada N., Karasawa T., Wakiya T. (2020). Iron overload as a risk factor for hepatic ischemia-reperfusion injury in liver transplantation: potential role of ferroptosis. Am J Transplant.

[229] Rothstein J.D. (2017). Edaravone: a new drug approved for ALS. Cell.

[230] Pang Y., Liu X., Wang X. (2022). Edaravone modulates neuronal GPX4/ACSL4/5-LOX to promote recovery after spinal cord injury. Front Cell Dev Biol.

[231] Tuo Q.Z., Liu Y., Xiang Z. (2022). Thrombin induces ACSL4-dependent ferroptosis during cerebral ischemia/reperfusion. Signal Transduct Targeted Ther.

[232] Xu S., Tuo Q.Z., Meng J., Wu X.L., Li C.L., Lei P. (2024). Thrombin induces ferroptosis in triple-negative breast cancer through the cPLA2α/ACSL4 signaling pathway. Transl Oncol.

[233] Miao Z., Tian W., Ye Y. (2022). Hsp90 induces Acsl4-dependent glioma ferroptosis via dephosphorylating Ser637 at Drp1. Cell Death Dis.

[234] Liu S., Gao Z., He W. (2022). The gut microbiota metabolite glycochenodeoxycholate activates TFR-ACSL4-mediated ferroptosis to promote the development of environmental toxin-linked MAFLD. Free Radic Biol Med.

[235] Yan H., Yan Y., Gao Y. (2022). Transcriptome analysis of fasudil treatment in the APPswe/PSEN1dE9 transgenic (APP/PS1) mice model of Alzheimer's disease. Sci Rep.

[236] Li C., Sun T., Jiang C. (2021). Recent advances in nanomedicines for the treatment of ischemic stroke. Acta Pharm Sin B.

[237] Xie J., Shen Z., Anraku Y., Kataoka K., Chen X. (2019). Nanomaterial-based blood-brain-barrier (BBB) crossing strategies. Biomaterials.

[238] Liao P., Wang W., Wang W. (2022). CD8^+^ T cells and fatty acids orchestrate tumor ferroptosis and immunity via ACSL4. Cancer Cell.

[239] Shu F., Shi Y., Shan X., Zha W., Fan R., Xue W. (2024). SIAH2-mediated degradation of ACSL4 inhibits the anti-tumor activity of CD8^+^ T cells in hepatocellular carcinoma. Crit Rev Eukaryot Gene Expr.

[240] Klett E.L., Chen S., Yechoor A., Lih F.B., Coleman R.A. (2017). Long-chain acyl-CoA synthetase isoforms differ in preferences for eicosanoid species and long-chain fatty acids. J Lipid Res.

[241] Teodoro B.G., Sampaio I.H., Bomfim L.H.M. (2017). Long-chain acyl-CoA synthetase 6 regulates lipid synthesis and mitochondrial oxidative capacity in human and rat skeletal muscle. J Physiol.

[242] Wang L., Qu F., Yu X. (2024). Cortical lipid metabolic pathway alteration of early Alzheimer's disease and candidate drugs screen. Eur J Med Res.

[243] Wen H., He Y., Tang Y. (2025). Altered immune response is associated with sex difference in vulnerability to Alzheimer's disease in human prefrontal cortex. Brain Pathol.

[244] Tu K., Zhou W., Kong S. (2024). Exploring biomarkers of Alzheimer's disease based on multi-omics and Mendelian randomisation analysis. Ann Hum Biol.

[245] Wu C.Y., Zhang Y., Howard P., Huang F., Lee R.H. (2025). ACSL3 is a promising therapeutic target for alleviating anxiety and depression in Alzheimer's disease. Geroscience.

[246] He H., Ji T., Lyu Y. (2025). BPDE induces ferroptosis in hippocampal neurons through ACSL3 suppression. Neurotoxicology.

[247] Long Q., Li T., Zhu Q., He L., Zhao B. (2024). SuanZaoRen decoction alleviates neuronal loss, synaptic damage and ferroptosis of AD via activating DJ-1/Nrf2 signaling pathway. J Ethnopharmacol.

[248] Gong K., Zhou S., Xiao L. (2025). Danggui Shaoyao San ameliorates Alzheimer's disease by regulating lipid metabolism and inhibiting neuronal ferroptosis through the AMPK/Sp1/ACSL4 signaling pathway. Front Pharmacol.

[249] Gao Y., Li J., Wu Q. (2021). Tetrahydroxy stilbene glycoside ameliorates Alzheimer's disease in APP/PS1 mice via glutathione peroxidase related ferroptosis. Int Immunopharmacol.

[250] Cheng L., Zhu X., Liu Y., Zhu K., Lin K., Li F. (2021). ACSL4 contributes to sevoflurane-induced ferroptotic neuronal death in SH-SY5Y cells via the 5' AMP-activated protein kinase/mammalian target of rapamycin pathway. Ann Transl Med.

[251] Abu-Elfotuh K., Mahran Y., Bayoumie El Gazzar W. (2025). Targeting ferroptosis/Nrf2 pathway ameliorates AlCl(3)-induced Alzheimer's disease in rats: neuroprotective effect of morin hydrate, zeolite clinoptilolite, and physical plus mental activities. Int J Mol Sci.

[252] Wang M., Xuan T., Li H., An J., Hao T., Cheng J. (2024). Protective effect of FXN overexpression on ferroptosis in L-Glu-induced SH-SY5Y cells. Acta Histochem.

[253] Peng W., Zhu Z., Yang Y. (2021). N2L, a novel lipoic acid-niacin dimer, attenuates ferroptosis and decreases lipid peroxidation in HT22 cells. Brain Res Bull.

[254] Hacioglu C., Kar F., Ozbayer C., Gundogdu A.C. (2024). *Ex vivo* investigation of betaine and boric acid function as preprotective agents on rat synaptosomes to be treated with Aβ (1-42). Environ Toxicol.

